# Prevalence of diabetes and hospitalization due to poor glycemic control in people with bladder cancer or renal cell carcinoma in Sweden

**DOI:** 10.1186/s12894-024-01536-2

**Published:** 2024-07-17

**Authors:** Emelie Andersson, Gunnar Brådvik, Fredrik O. L. Nilsson, Johannes Arpegård, Angela Strambi, Petter Kollberg, Katarina Steen Carlsson

**Affiliations:** 1 The Swedish Institute for Health Economics, Råbyvägen 2, 223 61 Lund, Sweden; 2 Pfizer AB, Solnavägen 3H, 113 63 Stockholm, Sweden; 3 Department of Surgical and Perioperative Sciences, Urology and Andrology, Umeå University, 901 85 Umeå, Sweden; 4 Department of Clinical Sciences, Malmö, Health Economics, Lund University, BMC F12, 221 84 Lund, Sweden; 5 Present address: Fondazione Toscana Life Sciences, Siena, Italy

**Keywords:** Genitourinary Cancer, Diabetes Mellitus Type 2, Immunomodulating treatment, Nested Case–Control Study, Population Registers, Urinary Bladder Neoplasms, Renal Cell Cancer

## Abstract

**Background:**

Bladder cancer (BC) and Renal cell carcinoma (RCC) are the most common urogenital cancers among both sexes, with a yearly global incidence of around 500 000 each. Both BC and RCC have been linked to diabetes. Poor glycemic control (malglycemia) is a serious consequence of diabetes and a possible consequence of systemic treatments used in BC and RCC. The objective of this study was to investigate the prevalence of diabetes and use of hospital-based care for malglycemia in people with BC or RCC.

**Methods:**

This Swedish retrospective population-based register study used national health-data registers for longitudinal data on cancer incidence covering 15 years, use of hospital-based health care, and filled prescriptions of outpatient medications. Study endpoints included co-prevalence of diabetes in individuals with BC/RCC, healthcare resource utilization due to malglycemia, use of systemic corticosteroids, and changes in diabetes management for people with concomitant type 2 diabetes.

**Results:**

We identified 36,620 and 15,581 individuals diagnosed with BC and RCC, respectively, between 2006 and 2019. The proportion of individuals registered with diabetes was 24% in BC and 23% in RCC. An association between BC/RCC and poor glycemic control was found, although the number of malglycemic events in hospital-based care were few (65/59 per 1000 individuals with diabetes and BC/RCC respectively with at least one event). An earlier switch to insulin-based diabetes management was observed in BC/RCC compared to matched individuals with type 2 diabetes but no cancer. The results also indicated an association between steroid treatment and poor glycemic control, and that systemic corticosteroids were more common among people with BC/RCC compared to diabetes controls.

**Conclusion:**

The high prevalence of diabetes and increased use of systemic corticosteroid treatment observed in this large national study highlights the need for specific clinical management, risk-assessment, and monitoring of individuals with BC/RCC and diabetes.

**Supplementary Information:**

The online version contains supplementary material available at 10.1186/s12894-024-01536-2.

## Introduction

Bladder cancer (BC) and renal cell carcinoma (RCC) are the two most common genitourinary cancers that affect both sexes. Globally, there are 570,000 new cases of BC and it accounts for 2% of all cancers. The corresponding numbers for RCC are 430,000 and 3% [1]. The highest rates have been observed in North America, Australia, and Europe [2].

Epidemiologic associations with diabetes mellitus have been shown for genitourinary cancers [3–5]. People with diabetes had a 50% increased risk of renal cancer regardless of other risk factors in a Swedish population-based retrospective cohort [6]. Links between bladder cancer risk and diabetes vary, with some data indicating associations [7–11]. However, concomitant prevalence of the illnesses in patients is common [12]. According to a population based cross-sectional analysis of cancer patients diagnosed within 3 years of the study, 8–18% of all patients had a coexisting diabetes diagnosis [13]. However, it is not clear whether the association stems from common risk factors such as obesity, ageing or use of specific drugs, or if there is a direct link between the diseases, or some combination [14, 15]. Nevertheless, the growing global prevalence of diabetes, with 537 million adults in 2021 [16], induces an increased focus on population-based research on prevalence and treatment patterns for BC, RCC and concomitant diabetes.

Diabetes in cancer patients is associated with a poorer quality of-life [17], higher all-cause mortality [4, 18] and higher hospitalization rate [19]. In patients with RCC specifically, a previous meta-analysis showed that the presence of both type 1 and type 2 diabetes was associated with poor overall and cancer-specific survival [20]. In patients with non-muscle invasive BC and diabetes a poor glycemic control is associated with disease progression and poor outcomes in terms of diabetes complications [21]. Moreover, people with cancer and diabetes may have a lower adherence to their diabetes medication [22]. This increases the risk for malglycemia, defined here as hypoglycemia, hyperglycemia or glycemic variability. Malglycemia is a serious condition related to an increased risk for diabetes complications and mortality as well as a lower quality of life [23, 24].

In recent decades targeted and immunomodulating cancer therapies such as the widely used anti-angiogenic tyrosine kinase inhibitors (TKI) and immune checkpoint inhibitors (ICI) have been introduced in the treatment of advanced stages of BC and RCC [25, 26]. For both classes of drugs, endocrine side effects have been reported [27]. Anti-angiogenic TKIs used in RCC may affect glucose metabolism and insulin signaling [27, 28], with both hypo- and hyperglycemia as reported side effects [29, 30]. ICIs may also impact the endocrine system through mechanisms that are currently not completely understood [27]. As a consequence, a new spectrum of immune-mediated adverse events including glycemic alterations, and even autoimmune type-1 diabetes, may affect cancer patients treated with ICI therapy [31]. Notably, ICI-induced toxicity is generally managed by administration of high dose corticosteroids [32, 33]. Beyond this role, systemic corticosteroids have a broad use in cancer treatment but can also increase the risk for hyperglycemia in people with and without diabetes [32]. Therefore, malglycemia may be worth specific attention in these cancer populations, whether as a comorbidity, a direct adverse event of a treatment or indirect consequence due to toxicity management. Studies investigating the magnitude of this problem and the related healthcare utilization are needed.

In Sweden, the large number of health data registers run by the National Board of Health and Welfare (NBHW) provides unique opportunities for longitudinal research. These highly qualitative registers with a long history constitute the foundation of a successful Swedish research tradition in the field of clinical epidemiology, and cover health data for the entire population. Combining data from these registers creates an opportunity to link incidence of cancer to health care resource utilization on an individual level.

The primary objective of this study was to investigate the co-prevalence of diabetes (either type 1 or type 2 diabetes) in people with BC or RCC in Sweden and health care resource utilization due to malglycemia. The secondary objective was to explore patterns of systemic corticosteroids use in people with BC or RCC, and changes in diabetes management for those with type 2 diabetes.

## Materials and methods

This is a retrospective, longitudinal, population-based register study. The study dataset included longitudinal data on cancer incidence covering 15 years, use of hospital-based health care (visits in outpatient specialist care and inpatient admissions), and filled prescriptions of outpatient medications. The main study period included incident cancers between 2006 and 2019, with additional retrospective information on diabetes diagnoses from 1997 and onwards. Observational individual-level data covered the period January 1, 2006 to December 31, 2020.

The study used three health data registers at the NBHW in Sweden: the Swedish Cancer Register (SCR), the National Patient Register (NPR), and the National Prescribed Drug Register (NPDR). It also used demographic information from the Register of the Total Population (RTB) at Statistics Sweden.

### Study population

This longitudinal study contains a study population of patients diagnosed with BC or RCC, respectively, as well as a sub-population diagnosed with type 1 or type 2 diabetes before cancer diagnosis together with matched controls. Data were record linked using personal identity numbers. A more detailed description of register sources and study database can be found in the Supplementary A. The persons were followed from the date they received their cancer diagnosis until end of the study period, emigration or death, which ever came first and then censored.

People with BC were included based on a registration in the SCR with a first time International Classification of Diseases and Related Health Problems version 10 (ICD-10) code C67 between 2006 and 2019. Correspondingly, the RCC population was defined as a first time ICD-10 code C64.

Diabetes together with its index date were defined as any registration of ICD-10 codes E10, E11 or E14 in inpatient or specialized outpatient care in the NPR (1997–2019) or filled prescriptions of glucose lowering medication (ATC code A10) in the NPDR (2005–2019). Using this information, a proportion of people with BC or RCC were classified with diabetes and given a diabetes index date. The subset of individuals that developed diabetes before they were diagnosed with cancer were matched 1:5 for control persons from a total diabetes population on year of birth, sex, and year of index date for diabetes. People with diabetes were further categorized as having type 1 or type 2 diabetes using information from the NPR and NPDR. More information on population selection criteria is reported in Supplementary B.

### Study endpoints

The study analyzed four endpoints (two primary and two secondary) for people with BC or RCC (cancer for short in the following). The primary endpoints were:

1. Co-prevalence of cancer and diabetes.
2. The incidence of poor glycemic control defined as the fraction of cancer cohorts with health care use related to malglycemia with reference to patterns in controls with diabetes but no cancer.

The co-prevalence of cancer and diabetes was defined as the fraction of the total population diagnosed with cancer years 2006–2019 that at the time of cancer diagnosis (also referred to as cancer index date) had, or subsequently developed diabetes during study follow-up. Health care use related to malglycemia was defined as hospitalizations or outpatient specialist visits with hypoglycemia or hyperglycemia as main or secondary diagnosis (ICD-10 codes E160, E161, E162 or R739). The primary endpoints are reported for the whole study population with cancer. The second primary endpoint is also reported for the subgroup of individuals with diabetes before they were diagnosed with cancer compared to matched diabetes controls.

The secondary endpoints of this study were:

3. The use of systemic corticosteroid treatment and its association with health care use related to malglycemia.
4. The change in diabetes management as measured by newly prescribed insulin in people with type 2 diabetes and cancer (type 1 diabetes need insulin by default).

The use of systemic corticosteroid was defined as ≥ 1 filled prescription of any drug with the ATC-code H02AB. Endpoint 3 is reported for the whole population of people with cancer. Endpoint 4 is reported for the subgroup of individuals with cancer and type 2 diabetes who were not using insulin at the time of diagnosis of cancer. Results were compared to that of matched diabetes controls.

### Statistical analysis

Baseline characteristics for all individuals were summarized using standard descriptive measures. This included number and proportion for categorical variables and mean with standard deviation, or median with interquartile range (IQR) for continuous variables. Endpoints were analyzed using the following methods:

The co-prevalence of cancer and diabetes (endpoint 1) was reported as the proportion with observed diabetes before the cancer index-date, as diabetes at any time during the study period, and as type 2 diabetes after the cancer diagnosis. The incidence of poor glycemic control (endpoint 2) was reported as rates per 1,000 individuals and rates per 10,000 person-years, for comparison between study groups and generalizability to other populations.

Endpoint 3, the use of systemic corticosteroid treatment and its association with health care use related to malglycemia, was described by the cumulative number of events per 1000 persons after first observed filled prescription of systemic corticosteroids following cancer diagnosis for BC or RCC with diabetes. This was compared to matched controls with diabetes but without cancer. Cox proportional hazard regression analysis was used to explore time to first hospitalization due to poor glycemic control after cancer diagnosis and its associations with systemic corticosteroid treatment. The analysis was performed controlling for sex, age, diabetes, and spread of cancer at diagnosis measured by presence of metastases according to the American Joint Committee on Cancer (AJCC) TNM staging system [34]. More information of the classification of malignant tumors can be found in Supplementary B.

The time to start of insulin-based diabetes treatment (endpoint 4) was analyzed in people with BC or RCC and type 2 diabetes and compared with matched controls with no cancer but with type 2 diabetes using Kaplan–Meier analysis. The equality of survivor functions was tested using a logrank test. Methodological considerations are included in Supplementary A.

A sensitivity analysis explored the impact on results of a broader inclusion of malignancies affecting the urinary tract (ICD 10 codes C66, C67, and C68), which did not alter the results. These results are available on request from the corresponding author.

Data were analyzed using STATA MC version 15.1 and R version 4.2.0 with the *survival* and *dplyr* packages.

## Results

The study population consisted of 36,620 individuals diagnosed with BC and 15,581 individuals diagnosed with RCC between 2006 and 2019. The national incidence of BC and RCC between 2006 and 2019 by gender, age-group and diabetes status are presented in Supplementary C Table S.1. More men than women had BC (75% men) and RCC (63% men). Descriptive statistics are presented in Table 1.

**Table 1:** Descriptive statistics

Characteristic	Bladder cancer	Renal cell carcinoma
Number of individuals	36,620	15,581
Total number of person-years after cancer index date	446,225	192,186
Follow up years		
Total population, min (max)	1 (15)	1 (15)
Total population, mean (SD)	5.5 (3.8)	5.6 (3.8)
Total population, median (Q25, Q75)	4 (2, 8)	5 (2, 8)
Diabetes before cancer diagnosis, mean (SD)	4.9 (3.4)	5.0 (3.5)
Diabetes controls, mean (SD)	6.0 (3.6)	6.6 (3.8)
Women, n (%)	9,265 (25)	5,758 (37)
Men, n (%)	27,355 (75)	9,823 (63)
Age at cancer diagnosis		
Mean (SD)	73.2 (10.8)	67 (11.8)
Median (25th percentile, 75th percentile)	74 (66.8, 81)	68.4 (60.1, 75.4)
Primary tumor at diagnosis (T), (%)		
T1	15 (< 1)	8,405 (54)
T2 + T3 + T4	7,252 (20)	5,641 (36)
Ta + Tis	17,853 (49)	3 (< 1)
Tx	559 (2)	357 (2)
Missing	10,941 (30)	1,175 (8)
Regional lymph nodes at diagnosis (N), n (%)		
N0	17,863 (49)	11,162 (72)
N1 + N2 + N3	1,140 (3)	1,329 (9)
Nx	1 (< 1)	0 (0)
Missing	17,616 (48)	3,090 (20)
Distant metastasis at diagnosis (M), n (%)		
M0	19,202 (52)	10,918 (70)
M1	1,192 (3)	2,467 (16)
Mx	14,322 (39)	1,279 (8)
Missing	1,904 (5)	917 (6)
Diabetes before cancer diagnosis, n (%)	6,191 (17)	2,665 (17)
Number of individuals with diabetes at any time n (%)	8,279 (23)	3,752 (24)
At least one filled prescription of steroids one year before cancer, n (%)^a^	3,441 (9)	1,752 (11)
Steroid treatment at any time 1st July 2005–2020	16,513 (45)	8,301 (53)
Registered date of death before study end, n (%)	17,383 (47)	6,649 (43)
Cause of death cancer, n (%)	10,493 (29)	4,626 (30)
Cause of death other, n (%)	6,890 (19)	2,023 (13)

^a^Based on persons diagnosed with cancer 1 July 2006–31 December 2019 (BC *n* = 35,495 and RCC *n* = 15,095) with ≥ 1 year of data on potential steroid medication available before cancer diagnosis

For BC, the total number of person-years between 2006 and 2020 was 446,225 of which 200,782 occurred after the cancer diagnosis (45%). Corresponding numbers for RCC was 192,186 person-years, of which 87,871 occurred after the cancer diagnosis (46%). The mean follow-up was 5.5 years for BC (IQR: 2 to 8 years) and 5.6 years for RCC (IQR: 2 to 8 years). The median follow-up was 4 years for BC and 5 years for RCC. Supplementary figures S.3 (BC) and S.4 (RCC) show population flowcharts for people with cancer and diabetes including exclusions due to emigration and the number of deaths observed in subgroups. Individuals with the same date registered for cancer diagnosis and death (BC *n* = 131, 0.4%; RCC *n* = 396, 2.5%) were censored in the time to event analyzes. In total, 19,237 persons with BC and 8,832 persons with RCC were alive and resident in Sweden at the end of the study period (31st of December 2020).

The proportion of individuals registered with diabetes at any time during the study period were similar among persons with BC (23%, *n* = 8,279) and RCC (24%, *n* = 3,752), as shown in Table 1. It was more common to be diagnosed with diabetes before the cancer diagnosis, with some differences between men and women. In the BC group, 18% of men and 12% of women had a registered diagnosis of diabetes before cancer diagnosis. For RCC, the corresponding numbers were 18 vs 16%. Baseline characteristics divided by gender are presented in Supplementary C, Table S.2 and S.3.

Resource utilization in outpatient and inpatient hospital-based care due to poor glycemic control is presented in Table 2. These results include visits or admissions with a main or secondary diagnosis associated with malglycemia registered after cancer index date for the study population and diabetes controls. The subgroup with cancer and diabetes included only individuals with pre-existing diabetes at the time of cancer index date. More information on individuals with diabetes diagnosis after cancer diagnosis is presented in Supplementary C Table S.4. The results in Table 2 show a significantly larger resource utilization of hospital-based care due to poor glycemic control among people with diabetes and cancer compared to diabetes controls. The number of individuals with at least one visit or admission due to poor glycemic control in hospital-based care per 1000 individuals with diabetes were 59 in the BC group and 65 in the RCC group. This can be compared with 47 and 52 per 1000 individuals for the diabetes controls of BC and RCC, respectively. Time to malglycemic event after cancer diagnosis for the BC and RCC group by diabetes and tumor status is further illustrated in Tables S.5/6 and figure S.5-S.10 in Supplementary C and D.

**Table 2:** Total number of contacts with outpatient and inpatient hospital-based care (visits or hospital admissions) with a main or secondary diagnosis associated with malglycemia after cancer diagnosis. All events observed in 2006–2020, after cancer index date and before emigration or death

**Statistics**	**Unit**	**BC with diabetes**^**a**^	**Control with diabetes**	***p*-value**^**b**^	**BC without diabetes**
BC study group	n (person-years)	6,178 (30,021)	23,565 (141,406)		28,340 (154,270)
≥ 1 visit or admission for malglycemia^c^	n per 1000 individuals (n)	59 (365)	47 (1,099)	< 0.001	2 (56)
Total number of visits or hospitalizations for malglycemia^c^	# per 1000 individuals (#)	96 (595)	72 (1,707)	< 0.001	3 (73)
	# per 10,000 person years (#)	198 (595)	121 (1,707)	< 0.001	5 (73)
**Statistics**	**Unit**	**RCC with diabetes**^**a**^	**Control with diabetes**	**p-value**^**b**^	**RCC without diabetes**
RCC study group	n (person-years)	2,659 (13,414)	11,203 (73,844)		11,827 (66,168)
≥ 1 visit or admission for malglycemia^c^	n per 1000 individuals (n)	65 (172)	52 (587)	0.014	3 (31)
Total number of visits or hospitalizations for malglycemia^c^	#per 1000 individuals (#)	107 (285)	81 (910)	< 0.001	3 (38)
	#per 10,000 person years (#)	212 (285)	123 (910)	< 0.001	6 (38)

n – number of individuals

^#^number of observations of visits or hospitalization. 660/194 individuals were first included in control group for BC/RCC and later observed with cancer index date (“switchers”). For these individuals, steroid treatment and malglycemic events are allocated according to their study group at the time of the observation. The table only covers the years during which the persons were alive and not emigrated

^a^Only include people with BC or RCC with registered diabetes diagnosis before cancer index date.

^b^From a two sided z-test.

^c^Main or secondary diagnosis

The association between poor glycemic control and different person characteristics, both at baseline and over time, were further explored in a Cox regression (see Table 3). As expected, having a diabetes diagnosis significantly increased the risk of experiencing an event of malglycemia requiring hospital-based care as main or secondary diagnosis for both the BC and RCC group. For RCC, a more advanced tumor status at diagnosis had a significant impact on malglycemic events. A similar pattern was observed for the BC group, but the association was not significant. The use of steroid treatment, captured by filled prescriptions of systemic corticosteroids, was significantly associated with events of poor glycemic control for patients with and without diabetes in both the BC and RCC group, as well as diabetes controls.

**Table 3:** Risk of malglycemic event after cancer index date by Cox regression analysis in full sample and in cancer with/without diabetes. Separate analyses for BC and RCC

	**BC total group**		**BC with diabetes**^**a**^		**BC control**	
	***N* = 36,620**		***N* = 6,178**		***N* = 23,565**	
	**Person years = 200,782**		**Person years = 30,021**		**Person years = 141,406**	
Variable	**Hazard ratio**	**95% CI**	**Hazard ratio**	**95% CI**	**Hazard ratio**	**95% CI**
Sex (ref: women)						
Men	0.99	0.80—1.22	0.77	0.60—0.99	1.06	0.91—1.24
M category^b^ (ref: M0)						
M1	1.36	0.70—2.65				
Mx or Missing	0.70	0.58—0.83				
Diabetes	14.83	12.08—18.20				
Steroid treatment	2.11	1.75—2.54	1.91	1.52—2.41	1.71	1.49—1.97
Age quartiles (ref: Age 18–59)						
Age 60–67	0.93	0.72—1.19	1.02	0.76—1.38	1.25	1.07—1.48
Age 68–74	1.42	1.11—1.80	1.47	1.11—1.94	1.59	1.35—1.87
Age > 74	1.48	1.15—1.91	1.61	1.19—2.22	1.94	1.63—2.30
	**RCC total group**		**RCC with diabetes**^**a**^		**RCC control**	
	***N* = 15,581**		***N* = 2,659**		***N* = 11,203**	
	**Person years = 87,871**		**Person years = 13,414**		**Person years = 73,844**	
Variable	**Hazard ratio**	**95% CI**	**Hazard ratio**	**95% CI**	**Hazard ratio**	**95% CI**
Sex (ref: women)						
Men	1.21	0.95—1.55	1.05	0.77—1.44	1.04	0.88—1.23
M category^b^ (ref: M0)						
M1	2.00	1.44—2.79				
Mx or Missing	0.87	0.62—1.23				
Diabetes	11.29	8.69—14.66				
Steroid treatment	2.68	2.10—3.43	2.06	1.49—2.85	1.73	1.44—2.09
Age quartiles (ref: Age 18–66)						
Age 67–73	0.88	0.62—1.22	1.02	0.67—1.54	1.00	0.79—1.26
Age 74–80	1.14	0.82—1.58	1.34	0.89—2.04	1.52	1.20—1.91
Age > 80	1.03	0.72—1.46	1.07	0.67—1.70	1.71	- 2.18

Age quartiles, and M category were measured at baseline, whereas the other variables changed over time in the regression

^a^Only include people with BC and RCC with registered diabetes diagnosis before cancer index date. Event related to malglycemia was defined as hospitalizations or outpatient specialist visits with hypoglycemia or hyperglycemia as main or secondary diagnosis (ICD-10 codes E160, E161, E162 or R739) from cancer index date to end of follow-up

^b^Distant metastasis at diagnosis

About half of the persons with cancer had been treated with steroids at any time during the study period, before or after cancer index date (see Table 1). Further analysis investigated steroid treatment patterns after cancer diagnosis in association with poor glycemic control, presented in Table 4 and in Figures S.11-S.13 in Supplementary D. The number of individuals per 1000 with at least one filled prescription of systemic corticosteroids after cancer index date were 458 in the BC group with diabetes compared with 247 among the matched diabetes controls. The corresponding figures were 405 for the RCC group compared with 256 for matched diabetes controls. The proportion of individuals with filled prescriptions of systemic corticosteroids were significantly larger among study persons with cancer and diabetes compared to diabetes controls. This was also the case for individuals with filled prescriptions of systemic corticosteroids and at least one visit or admission for malglycemia (24 and 36 per 1000 individuals in the BC and RCC groups with diabetes, respectively, compared with 17 and 20 per 1000 individuals in the control groups for BC and RCC, respectively). The number per 1000 individuals with malglycemic events within two years from systemic corticosteroid treatment after cancer index date were 11 in the BC group with diabetes and 15 in the RCC group with diabetes, which was significantly larger compared to diabetes controls with systemic corticosteroid treatment.

**Table 4:** Filled prescriptions of steroids after cancer index date and the association with malglycemia. All events 2006–2020 after cancer index date

Statistics	Unit	Cancer with diabetes^a^	Control with diabetes	*p*-value^b^	Cancer without diabetes
**BC study group**	n	6,178	23,565		28,340
≥ 1 filled prescription of steroids after cancer index date^c^	n per 1,000 individuals (n)	458 (2,831)	247 (5,815)	< 0.001	341 (9,654)
≥ 1 filled prescription of steroids after cancer index date and ≥ 1 visit or admission for malglycemia^c,d^	n per 1,000 individuals (n)	24 (151)	17 (392)	< 0.001	1 (25)
Number of individuals with malglycemia^d^ within two years of steroid treatment after cancer index date	n per 1000 individuals (n)	11 (71)	6 (149)	< 0.001	0 (0)
**RCC study group**	n	2,659	11,203		11,827
≥ 1 filled prescription of steroids after cancer index date^c^	n per 1,000 individuals (n)	405 (1,090)	256 (2,866)	< 0.001	421 (4,974)
≥ 1 filled prescription of steroids after cancer index date and ≥ 1 visit or admission for malglycemia^c,d^	n per 1,000 individuals (n)	36 (95)	20 (224)	< 0.001	2 (25)
Number of individuals with malglycemia^d^ within two years of steroid treatment after cancer index date	n per 1,000 individuals (n)	15 (39)	7 (81)	< 0.001	1 (0)

^a^Only include people with BC and RCC with registered diabetes diagnosis before cancer index date.

^b^From a two sided z-test

^c^Observed events from cancer index date to end of follow-up

^d^Main or secondary diagnosis

**Fig. 1:**
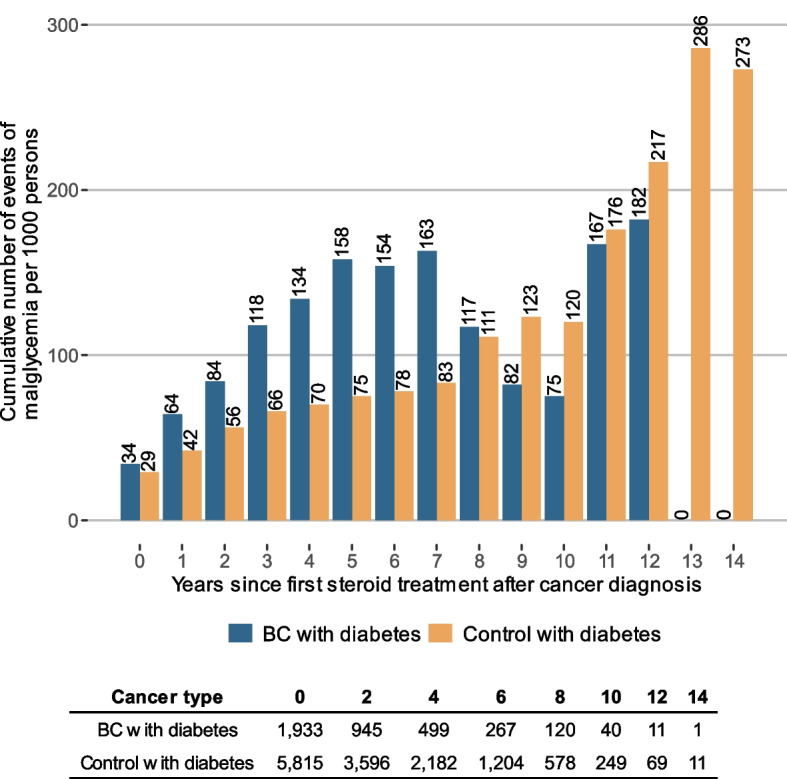
Cumulative number of malglycemic events per 1,000 persons (prevalence) after first observed filled prescription of steroids after cancer diagnosis among persons with BC. The events shown in the figure are weighted to represent events per 1,000 persons fulfilling these criteria and still in follow-up in the given year

**Fig. 2:**
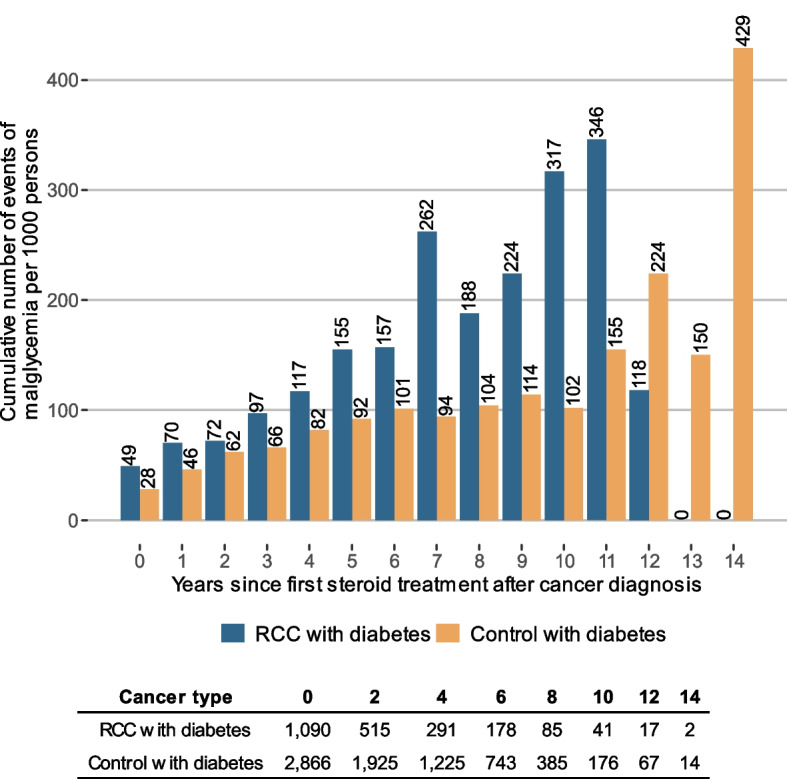
Cumulative number of malglycemic events per 1,000 persons (prevalence) after first observed filled prescription of steroids after cancer diagnosis among persons with RCC. The events shown in the figure are weighted to represent events per 1,000 persons fulfilling these criteria and still in follow-up in the given year

Cumulative events of malglycemia registered in hospital-based care after cancer index date and filled prescriptions of systemic corticosteroids are presented in Figs. 1 and 2. The cumulative events are presented for the BC and RCC group with diabetes together with diabetes controls for the study period. The decreasing number at risk during the study period is due to limited follow-up for some individuals and time of death. Each bar represents the cumulative number of malglycemic events per 1000 individuals still in follow-up at that specific time. The figures illustrate a higher rate of malglycemic events within the first years after systemic corticosteroid treatment for people with diabetes and cancer compared to diabetes controls. There is a shift during the last years of follow-up where the cumulative events of malglycemia were higher among diabetes controls. This is probably due to few individuals remaining in the cancer group, which is illustrated by the number at risk.

**Fig. 3:**
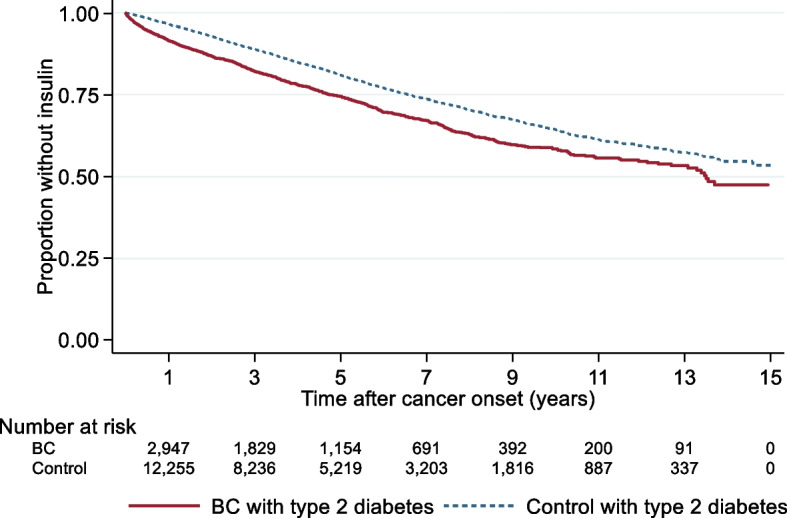
Risk of start of insulin treatment for people with a BC diagnosis from Jan 1, 2006 to Dec 31, 2019 and type 2 diabetes before cancer diagnosis compared to controls with type 2 diabetes but no BC-diagnosis. Kaplan–Meier analysis. Log-rank test *p* < 0.001

**Fig. 4:**
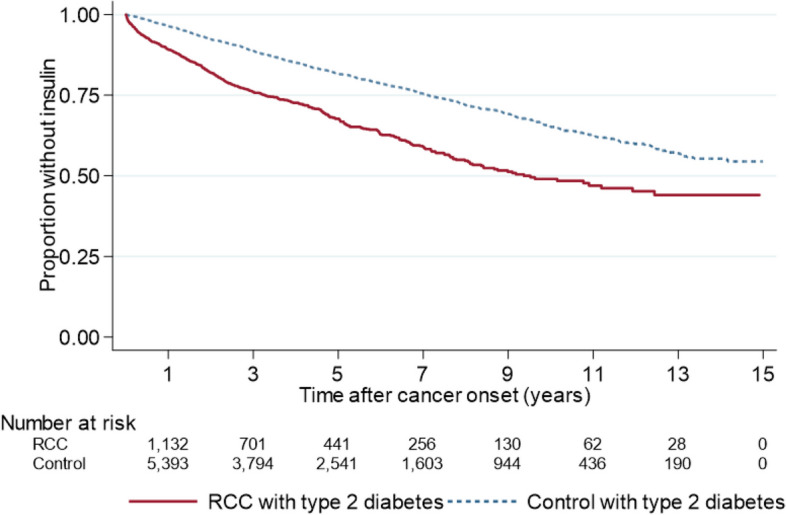
Risk of start of insulin treatment for people with a RCC diagnosis from Jan 1, 2006 to Dec 31, 2019 and type 2 diabetes before cancer diagnosis compared to controls with type 2 diabetes but no RCC-diagnosis. Kaplan–Meier analysis. Log-rank test *p* < 0.001

Figures 3 and 4 present survival time analyzes of time to start of insulin-based therapy for patients with type 2 diabetes and BC or RCC. People with cancer and type 2 diabetes were compared with type 2 diabetes controls. The analysis started at the diagnosis of cancer for the cases and only include individuals with type 2 diabetes with no insulin treatment at that time. Two other study setups are included in Supplementary D: time from 1997 with first observed diabetes (Figure S.14-S.15) and starting on July 1, 2005 when the Swedish prescription register where introduced (Figure S.16-S.17). The results show a significantly faster increase in the proportion with insulin treatment over time among individuals with cancer and type 2 diabetes compared with type 2 diabetes controls.

## Discussion

Findings from previous studies have suggested that cancer can lead to poorer diabetes-related outcomes as well as vice versa [35, 36]. There are several underlying mechanisms that have been proposed to explain the relationship between diabetes and cancer. Hyperglycemia, insulin resistance, elevated insulin, and insulin-like growth factor-1 (IGF-1) levels, inflammatory cytokines, dyslipidemia, increased leptin, and decreased adiponectin have all been attributed to the increased risk of cancer in patients with diabetes [37]. Further oxidative stress through the production of superoxide and reactive oxygen species (ROS) [38, 39] strongly influences the expression of genes and signal transduction pathways that play a crucial role in tumor development [40]. These factors can affect gene expression and signaling pathways involved in tumor growth, as well as interfere with cell proliferation and apoptosis [41] all of which may contribute to a poorer cancer prognosis and outcome.

The increased risk of a worsened diabetes outcome following a cancer diagnosis may partly be attributed to cancer-management interventions. Corticosteroids reduces insulin sensitivity which may cause aggravating hyperglycemia, and chemotherapeutic agents, such as L-asparginase, directly inhibits insulin release. Further, direct cancer effects, such as cancer cachexia is associated with increased insulin resistance and impaired glucose tolerance, and cachexia-associated cytokines, such as tumor necrosis factor and interleukin- 6 are associated with the development of insulin resistance [42].

The proportion of individuals registered with diabetes was 23 and 24% respectively in the BC and RCC Swedish population of this study. As a reference, the prevalence of diabetes in the general population in Sweden is about 7% among adults and 12% among people 65 years and older [43–45]. A higher prevalence of diabetes among people with BC and RCC has also been shown in previous studies from Italy and the US [11, 46].

Furthermore, the prevalence of diabetes at diagnosis of both BC and RCC was higher in men compared to women in this study. This seems in line with a higher prevalence of diabetes among males in Sweden, with female representing about 42% to 46% of the total diabetes population [4, 45]. The small difference observed here in the RCC study sample (18 in men and 16% in women) is in line with the proportions reported for the general population. However, previous studies on RCC have reported a higher prevalence of diabetes among women compared to men [46]. Diabetes in women was also reported to confer a significantly greater risk of kidney cancer relative to men [5]. The difference in diabetes occurrence observed for the BC population in this study (18% in men and 12% in women) seems not to be a reflection of the difference in the gender distribution of diabetes in Sweden, pointing possibly to other reasons.

An association between BC/RCC and poor glycemic control was found in this study, although the number of malglycemic events in hospital-based care were few in absolute numbers. The number of events per 1000 individuals was 96 in the BC group with diabetes and 107 in the RCC group with diabetes. The low numbers may indicate that malglycemic events leading to hospital-care is relatively uncommon among patients with BC or RCC. Still, events of poor glycemic control can be serious and even life threating and therefore deserving close attention and management, especially in the context of frail cancer patient populations. A lower adherence to diabetes medication in people with both diabetes and cancer [22] might also have influenced the outcome in terms of malglycemia.

Information on escalation of diabetes management in terms of insulin therapy can increase the understanding of health care utilization due to poor glycemic control over disease duration and time. The results from this study showed that the proportion with insulin treatment increased significantly faster over time among individuals with cancer and type 2 diabetes compared with type 2 diabetes controls. Initiation of insulin-based therapy among individuals with type 2 diabetes may be an indication of poorer glycemic control.

A considerable and increasing proportion of BC and RCC patients receive systemic therapies in the context of their cancer that can directly and indirectly impact glucose and insulin metabolism. Therefore, they may require specific clinical management, risk-assessment and monitoring in this regard. The results also indicated an association between steroid treatment and poor glycemic control. Since a larger share of the BC/RCC group have used steroid treatment compared to controls, this might fully or partially explain the higher prevalence of poor glycemic control among individuals with cancer.

This study used national individual-level data with high coverage [47] and a long follow-up of 15 years, which gives a solid base for analyzing healthcare resource utilization and patient pathways. The study data represent broad BC and RCC populations which include patients in all phases of the disease. This complements studies focusing on patients at later stages of the disease. Data show that people with BC or RCC and concomitant diabetes have higher health care utilization for treatment of malglycemia compared with people with diabetes and no cancer. A limitation of the study data is the lack of information on less severe malglycemic events not requiring hospital-based care and data on steroid treatment used within inpatient care. This may possibly have underestimated the incidence of malglycemia in the study population, but it is likely that most severe cases are captured.

Cancer patients with co-prevalent diabetes are an important group from a health care planning perspective as it will likely increase in the future. Whether or not the positive association with diabetes and the risk of several cancer forms is genuine [15], a growing prevalence of diabetes in the general population [48] implies increased number of people at risk of concomitant disease. Moreover, the incidence of many cancer forms is increasing partly due to screening, improved diagnostics, and an aging population. Thus, it is important to expand the knowledge about patients with concomitant cancer and diabetes including the healthcare utilization linked to the management of this specific patient population.

The present study focused on BC and RCC, two genitourinary malignancies affecting both females and males. These are among the cancer diagnoses where an increasing proportion of patients are getting exposed to oncological treatment that may deserve specific management and attention in the context of a comorbidity such as diabetes. In addition to systemic corticosteroids that cancer patients may often receive, anti-angiogenic TKIs (RCC) and ICIs (both BC and RCC) can, directly or indirectly, impact glycemic equilibrium. In particular, ICIs have recently been introduced in the adjuvant setting for both BC and RCC, expanding the proportion of patients getting the chance to obtain a clinical benefit but also, inevitably, to experience undesired consequences on glycemic control. This study provides prevalence estimates of the Swedish BC and RCC patient populations with diabetes and the impact of malglycemic events on healthcare consumption for these groups. Its time frame mainly consists of the period before the extensive use of targeted and immunomodulating cancer therapies.

A high prevalence of diabetes in individuals with BC or RCC together with increased use of systemic corticosteroid treatment and higher risk for poor glycemic control compared with diabetes controls were observed in this large national study. This highlights the need for specific clinical management, risk-assessment, and monitoring of individuals with BC or RCC, and concomitant diabetes in order to decrease the risk of poorer diabetes and cancer-related outcomes.

### Supplementary Information

Supplementary Material 1.

## Data Availability

Subject-level data from national registers hosted by Swedish national authorities are available for research after formal evaluation of the research protocol by the Swedish Ethical Review Authority and by the respective national authorities providing data. Permission to conduct research are granted on a case by case basis to a limited number of named persons.

## Abbreviations

BC: Bladder cancer
RCC: Renal cell carcinoma
TKI: Anti-angiogenic tyrosine kinase inhibitors
ICI: Immune checkpoint inhibitors
NBHW; Socialstyrelsen: The National Board of Health and Welfare
SCR: The Swedish Cancer Register
NPR: The National Patient Register
NPDR: The National Prescribed Drug Register
RTB: The Register of the Total Population
ICD-10: International Classification of Diseases and Related Health Problems version 10
IQR: Interquartile range
AJCC: The American Joint Committee on Cancer
