# Supplementary material for: Prevalence of diabetes and hospitalization due to poor glycemic control in people with bladder cancer or renal cell carcinoma in Sweden
Source: BMC Urol. 2024 Jul 17;24:148. doi: 10.1186/s12894-024-01536-2 (PMC11253448; doi:10.1186/s12894-024-01536-2)
Supplement: Supplementary file 1 — Supplementary Material 1. [file 12894_2024_1536_MOESM1_ESM.docx]

# Prevalence of diabetes and hospitalization due to poor glycemic control in people with bladder cancer or renal cell carcinoma in Sweden

Emelie Andersson, Gunnar Brådvik, Fredrik Nilsson, Johannes Arpegård, Angela Strambi, Petter Kollberg, Katarina Steen Carlsson

Contents

[Prevalence of diabetes and hospitalization due to poor glycemic control in people with bladder cancer and renal cell carcinoma in Sweden 1](#_Toc162440765)

[Supplementary A Description of register sources and study database 3](#_Toc162440766)

[Supplementary B Population selection criteria 7](#_Toc162440767)

[Renal Cell Carcinoma, RCC 7](#_Toc162440768)

[Bladder cancer, BC 7](#_Toc162440769)

[Diabetes 7](#_Toc162440770)

[Controls for (sub)Dataset B 7](#_Toc162440771)

[Categorization of type 1 diabetes and type 2 diabetes 8](#_Toc162440772)

[Classification of Malignant Tumors 8](#_Toc162440773)

[References 8](#_Toc162440774)

[Supplementary C Result tables 9](#_Toc162440775)

[Supplementary D Result figures 17](#_Toc162440776)

[Glycemic events 17](#_Toc162440777)

[Filled prescriptions of steroids 23](#_Toc162440778)

[Insulin treatment 26](#_Toc162440779)

Figures

[Figure S1 Study population and sources of data 3](#_Toc162343521)

[Figure S2 Dataset A (all people with RCC and BC) and dataset B (people with diabetes at the time of RCC or BC diagnosis) with its controls with diabetes but no RCC or BC matched on year of birth, sex and index date for diabetes. 4](#_Toc162343522)

[Figure S3 Flow chart dataset renal cell carcinoma. 5](#_Toc162343523)

[Figure S4 Flow chart bladder cancer dataset 6](#_Toc162343524)

[Figure S5 Kaplan Meier estimation of time to first glycemic event leading to a hospital visit or admission after diagnosis of BC and RCC with and without diabetes. 17](#_Toc162343525)

[Figure S6 Kaplan Meier estimation of time to first glycemic event leading to a hospital visit or admission from diagnosis of BC and RCC with diabetes 18](#_Toc162343526)

[Figure S7 Kaplan Meier estimation of time to first glycaemic event leading to a hospital visit or admission after diagnosis of RCC stratified by status for metastasis at diagnosis. 19](#_Toc162343527)

[Figure S8 Kaplan Meier estimation of time to first glycaemic event leading to a hospital visit or admission after diagnosis of BC stratified by status for metastasis at diagnosis. 20](#_Toc162343528)

[Figure S9 Kaplan Meier estimation of time to first glycaemic event leading to a hospital visit or admission from diagnosis of RCC with diabetes stratified by status for metastasis at diagnosis. 21](#_Toc162343529)

[Figure S10 Kaplan Meier estimation of time to first glycaemic event from diagnosis of BC with diabetes stratified by status for metastasis at diagnosis. 22](#_Toc162343530)

[Figure S11 Time to first filled prescription of steroids after diagnosis of BC and RCC. 23](#_Toc162343531)

[Figure S12 Kaplan Meier estimation of time to first filled prescription of steroids from diagnosis of RCC stratified by registered M-status according to TNM staging system on cancer index date. 24](#_Toc162343532)

[Figure S13 Kaplan Meier estimation of time to first filled prescription of steroids from diagnosis of BC stratified by registered status of radiologically shown metastasis on cancer index date. 25](#_Toc162343533)

[Figure S14 Kaplan-Meier survival curves for start of insulin treatment for people with type 2 diabetes and later RCC diagnosis compared to matched controls with type 2 diabetes only. Analysis time from first observed diabetes after 1 January 1997. RCC diagnosis from Jan 1, 2006 to Dec 26](#_Toc162343534)

[Figure S15 Kaplan-Meier survival curves for start of insulin treatment for people with type 2 diabetes and later BC diagnosis compared to matched controls with type 2 diabetes only. Analysis time from first observed diabetes after 1 January 1997. RCC diagnosis from Jan 1, 2006 to Dec 27](#_Toc162343535)

[Figure S16 Kaplan-Meier survival curves for start of insulin treatment for people with diagnosis of RCC and type 2 diabetes before cancer diagnosis. Controls have same year of diabetes diagnosis as cancer cases. Observation time starts Jul 1, 2005. 28](#_Toc162343536)

[Figure S17 Kaplan-Meier survival curves for start of insulin treatment for people with diagnosis of BC and type 2 diabetes before cancer diagnosis. Controls have same year of diabetes diagnosis as cancer cases. Observation time starts Jul 1, 2005. 29](#_Toc162343537)

Tables

[Table S1 National incidences of RCC and BC 2006-2019. Total number of persons and number and proportion of women per year, of people aged >70 years old at cancer diagnosis and with diabetes on cancer index date. 10](#_Toc162343247)

[Table S2 Baseline characteristics. Men only. 11](#_Toc162343248)

[Table S3 Baseline characteristics. Women only. 12](#_Toc162343249)

[Table S4 Individuals categorised with type 2 diabetes and with diabetes diagnosis after cancer diagnosis. 13](#_Toc162343250)

[Table S5 Number of glycaemic events for people with RCC and diabetes, controls with diabetes but no RCC, and for RCC without diabetes. Results by 1,000 individuals and by 10,000 person years. All events and period after cancer index date and all events after diabetes index date. 14](#_Toc162343251)

[Table S6 Number of glycemic events for people with BC and diabetes, controls with diabetes but no BC, and for BC without diabetes. Results by 1,000 individuals and by 10,000 person years. All events and period after cancer index date and all events after diabetes index date. 16](#_Toc162343252)

# Supplementary A Description of register sources and study database

This study dataset combined information from three health data registers at the National Board of Health and Welfare in Sweden: the Swedish Cancer Register (SCR), the National Patient Register (NPR), and the National Prescribed Drug Register (NPDR), and demographic information from the Register of the Total Population (RTB) at Statistics Sweden. The National Patient Register (NPR) covers inpatient care episodes and outpatient visits, including information of medical procedures and diagnosis codes. The Swedish Cancer Register (SCR) (since 1958) contains tumor and pathology data from all patients diagnosed with cancer in Sweden. The National Cause of Death Register (CDR) (1961) contain information on the cause of death. From July 2005, the Prescribed Drug Register (PDR) covers date and amount of medication prescribed and dispensed to the patient. Briefly, Figure S1 illustrates the population selected for this study consisting of all incident cases of RCC and BC (light blue box) registered in the SCR 2000-2019. It was expected that there would be about 24,000 people with RCC and about 60,000 people with BC based on reported annual incidence in Sweden. The NBHW retrieved additional information on people with diabetes based on registered diabetes diagnosis in the NPR (1997-2019) and/or filled prescriptions of glucose lowering medication (ATC-code A10) in the SPDR (2005-2019; the white box). The full population of people with diabetes formed an interim dataset which was used internally at the NBHW and not included in the delivery to the research team in its entirety. It was expected that the two patient groups overlap as illustrated by the dashed blue and white rectangle based on reported excess risk of RCC and BC for people with diabetes.


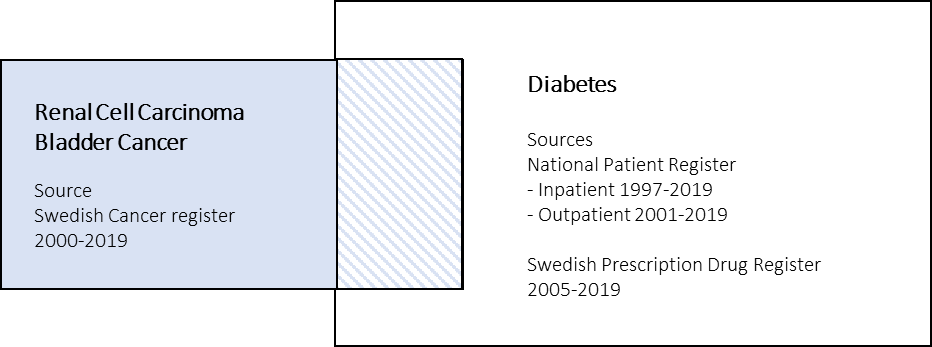


Figure A.1 Study population and sources of data

Figure S1 Study population and sources of data

People with RCC and BC – Dataset A – formed the main population. Dataset A was used to address primary and secondary objectives on prevalence of diabetes, incidence of hospital-based care due to poor glycemic control and use of selected medications. People with diabetes at the time of diagnosis of either RCC or BC (dashed area in Figure S1) was studied as a pre-defined subgroup – Dataset B. The NBHW in collaboration with Statistics Sweden added matched controls from the total diabetes population (white box in Figure S1) to people in Dataset B. Controls were matched 5:1 based on year of birth, sex, and index date for diabetes in the NBHW diabetes population.


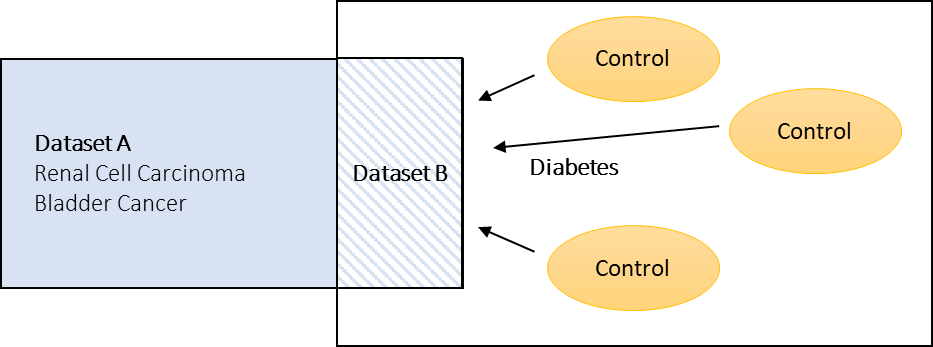


Figure S2 Dataset A (all people with RCC and BC) and dataset B (people with diabetes at the time of RCC or BC diagnosis) with its controls with diabetes but no RCC or BC matched on year of birth, sex and index date for diabetes.

The analyses of study endpoints were carried out in Dataset A as a whole and for the subgroup Dataset B. They encompassed comparisons of outcomes for Dataset B with i) matched controls among people with diabetes (but no RCC or BC); and ii) people in Dataset A but not in Dataset B.

We have carefully considered the application of tools that handle potential competing risks, e.g. using Fine Gray instead of Cox regression and Kaplan-Meier survival curves. However, there seems to be limitations in the library in R, cmprisk (written by Bob Gray), which used in combination with the survival library in R, to preform Fine Gray as well as limitations in Stata to handle central aspects of the respective research questions.

1. The analysis of the effect of steroids on malglycemic events uses binary time-varying covariate to allow steroid treatment to start at different points in time, if ever. Time-varying binary covariate works fine with the Cox regression analysis in the R library survival. However, the inclusion of binary time-varying covariate is, to the best of our understanding, not supported by the R-libraries cmprisk and survival. As the impact of use of steroids on malglycemic events is a key interest, and we are keen to keep the cancer onset (matching date for BC and RCC controls) as starting point in order to include all individuals and not only those on steroids, we do not seem to be able to produce results by Fine-Gray competing risk with the tools at hand.
2. The analysis of the time to insulin treatment for people with cancer and type 2 diabetes compared to people with only type 2 diabetes fails too when estimated with stcrreg in Stata v15.0. We wanted our design to not make the mistake of conditioning inclusion/exclusion in the dataset on future events (i.e cancer onset after diabetes onset). The dataset therefore allows people selected initially as controls (that is with type 2 diabetes but no cancer) to switch status if they are later diagnosed with either of the studied forms of cancer. Apparently, this switching causes the stcrreg to generate the error message that it does not support multiple failures. We believe this may be due to the fact that the competing risk models keep individuals who fail in the risk set. We do not seem to be able to incorporate both our wish not to condition of future events when selecting controls and to produce competing event analyses with stcrreg. With our setup we allow individuals to change group assignment. The same error message is not produced for either Kaplan Meier or Coxregression with the same stset sample, probably as censored individuals exit from the analysis. What happens when a control is diagnosed with cancer is that s/he gets a new start date. While being a control, s/he has the same start date as her/his case. When becoming a case, it is her/his own cancer onset day that is the start date. It would have been interesting to explore to what extent the competing risk perspective would have altered the conclusion as regards there seemingly being a difference in proportion of people with insulin treatment for their type 2 diabetes between those with cancer as well and controls (type 2 diabetes without cancer). Unfortunately, it does not seem to be possible with the currently available tools and definitions.


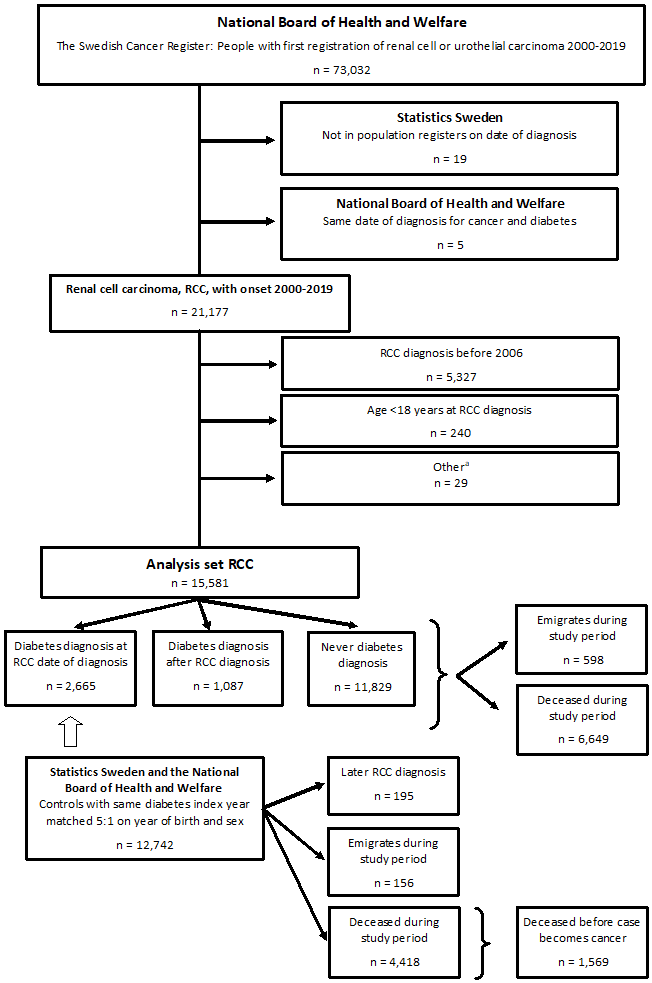


Figure S3 Flow chart dataset renal cell carcinoma.

1. Non-resident or emigration during year of diagnosis (14), T-status zero (13), and other (2)


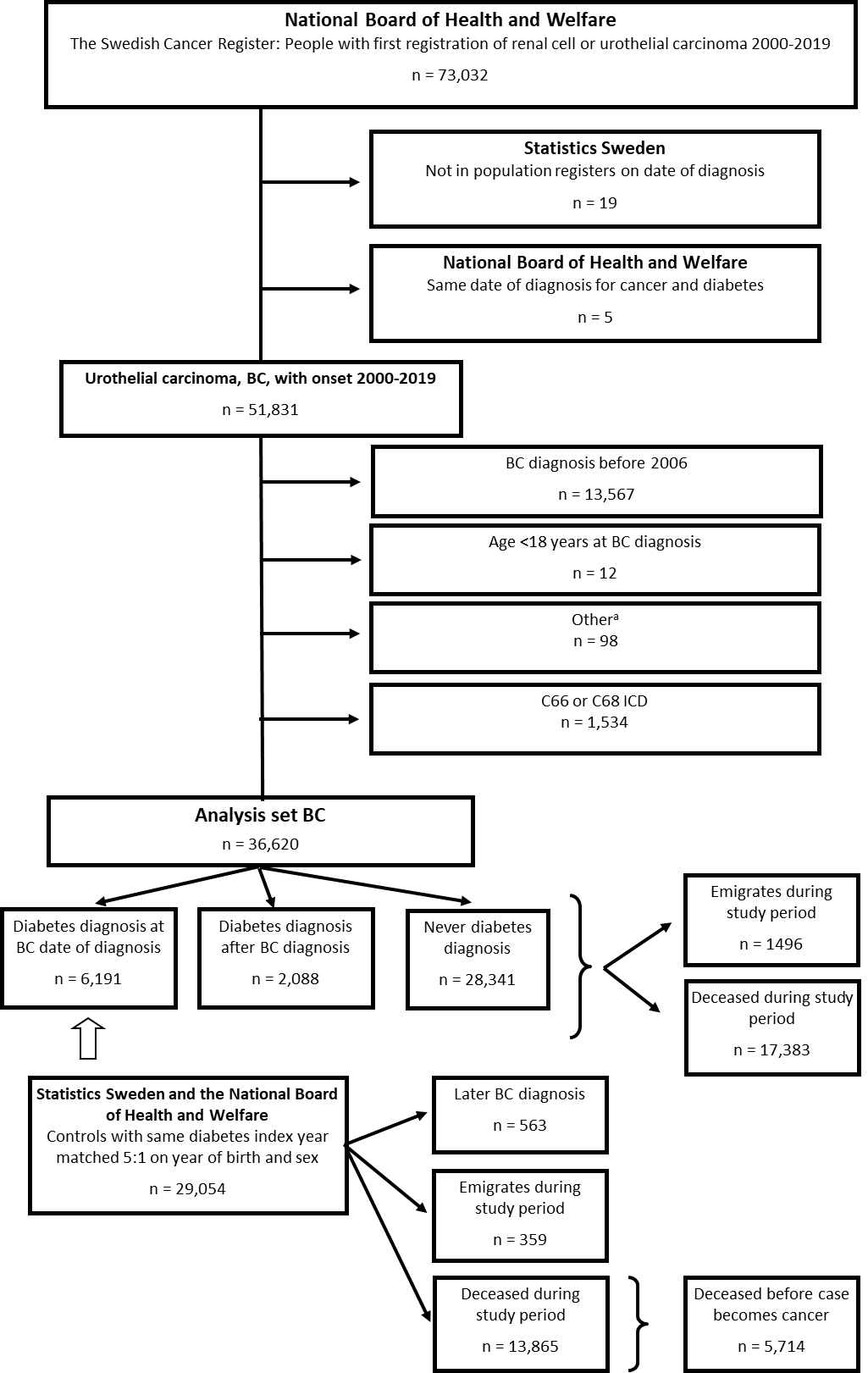


Figure S4 Flow chart bladder cancer dataset

1. Non-resident or emigration during year of diagnosis (62), T-status zero (33), and other (3)

# Supplementary B Population selection criteria

## Renal Cell Carcinoma, RCC

People with RCC include all individuals who received a diagnosis of RCC registered in the SCR between 1 January 2006 and 31 December 2019. All individuals with a registration of International Classification of Diseases and Related Health Problems version 10 (ICD-10) code C64 was included in Dataset A. Each individual was assigned an RCC index date which is defined as the first occurrence of a registration of RCC in the SCR. The total number of individuals was 15,581 with a total of 192,186 person-years in 2006-2020 including the years before cancer index date.

## Bladder cancer, BC

People with BC include all individuals who received a diagnosis of BC registered in the SCR between 1 January 2006 and 31 December 2019. All individuals with a registration of ICD-10 codes C66, C67 or C68 was included in Dataset A. Each individual was assigned a BC index date which is defined as the first occurrence of a registration of BC in the SCR. The total number of individuals was 36,620 with a total of 446,225 person-years in 2006-2020 including the years before cancer index date.

## Diabetes

NBHW retrieved an interim population of people with diabetes (white box in Figure S2) based on at least one of the following selection criteria:

- at least one health care visit or inpatient stay with diabetes as main or secondary diagnosis including E10 (type 1 diabetes), E11 (type 2 diabetes), or E14 (unspecified diabetes) in any year in the NPR from 1997 to 2019, and/or
- at least two dispensed prescriptions of glucose lowering medication (ATC-codes A10) with dispense dates not more than six months apart in the NPDR from July 1, 2005 to December 31, 2019 (or latest available data). Each individual was assigned a diabetes index date which is defined as the first occurrence of either criterion A or criterion B, whichever happens first.

Women with gestational diabetes are captured by these criteria if they use glucose lowering medication. While gestational diabetes is associated with an increased risk of later development of type 2 diabetes as shown for instance in a Swedish register-based study [[1](#_ENREF_1)], it is not in itself a lifelong chronic condition. The NBHW excluded women with gestational diabetes (ICD-10 O24) treated with glucose lowering medication temporarily from the study population. This may be operationalized as a limit of the exclusion from eligibility up to 2 years after the child is born. If the woman again meets inclusion criteria as stated above after that, she should be included with the index date of this second occurrence, provided it is not a repeated case of gestational diabetes in a new pregnancy.

## Controls for (sub)Dataset B

For people with RCC and BC in Dataset B, a comparison group was constructed, drawn from the overall diabetes population previously described. These non RCC/BC diabetes controls were matched 5:1 on diabetes index year, year of birth and sex respectively, to individuals in Dataset B. Further, controls may not have a history of RCC or BC at the time of matching and were selected without reversal.

## Categorization of type 1 diabetes and type 2 diabetes

The study database contains information on diabetes related use of hospital-based health care and filled prescriptions of glucose lowering medications used in the diabetes management. This information was used to construct a registry-based categorization of people with diabetes into type 1 diabetes (T1D) and type 2 diabetes (T2D). This study applied the same principles as was used in two recent publications [[2](#_ENREF_2), [3](#_ENREF_3)]. Briefly, the following principles was applied:

- T1D: People with hospital visits and admissions with ICD code E10 (T1D) but no registration of E11 (T2D) or E14 (Unspecified diabetes) were considered as clear cases of T1D.
- T2D: People with hospital visits and admissions with ICD code E11 (T2D) or E14 (Unspecified diabetes) but no registration of E10 were considered as clear cases of T2D.

Information on type of glucose lowering medication in the SPDR was used to further separate T1D from T2D. People with insulin (ATC code A10A) could be either T1D or T2D. People with at least one filled prescription of ATC code A10B (other glucose lowering medications) was considered as an indication of T2D. Remaining uncertain cases were classified as type 2 diabetes, although it is likely that some of these have type 1 diabetes. Classification of type 1 and type 2 diabetes were only used in the analysis of changes in diabetes management measured by newly prescribed insulin in people with type 2 diabetes. This included individuals with type 2 diabetes who were not using insulin at the time of diagnosis of cancer and therefor it is unlikely that these individuals have type 1 diabetes.

## Classification of Malignant Tumors

In order to classify the tumor, the node and the metastasis at the date of the cancer diagnosis, we used the TNM Classification of Malignant Tumors (TNM). In the TNM system, the cancer overall stage is determined by a description of the tumor (T), the node (N) and the metastasis (M) [[4](#_ENREF_4)]. These three description categories are based on the size of the tumor, the regional lymph node involvement of the tumor and the presence of distant metastases of the primary tumor.

In the cases where T, N or M was not assessed at the cancer diagnosis, this is marked as Tx, Nx or Mx respectively.

## References

1. Anderberg, E., K.S. Carlsson, and K. Berntorp, *Use of healthcare resources after gestational diabetes mellitus: a longitudinal case-control analysis.* Scand J Public Health, 2012. **40**(4): p. 385-90.

2. Andersson, E., et al., *Costs of diabetes complications: Hospital based care and absence from work for 392,200 people with type 2 diabetes and matched controls in Sweden.* Diabetologia, 2020.

3. Persson, S., et al., *Days absent from work as a result of complications associated with type 2 diabetes: Evidence from 20 years of linked national registry data in Sweden.* Diabetes, Obesity and Metabolism, 2020.

4. Rosen, R.D. and A. Sapra, *TNM Classification*, in *StatPearls*. 2022: Treasure Island (FL).

# Supplementary C Result tables

Table S1 National incidences of RCC and BC 2006-2019. Total number of persons and number and proportion of women per year, of people aged >70 years old at cancer diagnosis and with diabetes on cancer index date.

| **Year** | **Renal cell carcinoma, n (%)** | | | | | **Bladder cancer, n (%)** | | | |
| --- | --- | --- | --- | --- | --- | --- | --- | --- | --- |
|  | **Total** | **of which** | | | | **Total** | **of which** | | |
|  |  | **Women** | **Aged>70y** | **Diabetes** |  | | **Women** | **Aged>70y** | **Diabetes** |
| 2006 | 965 | 402 (42) | 395 (41) | 143 (15) | 2,221 | | 533 (24) | 1,350 (61) | 269 (12) |
| 2007 | 988 | 390 (39) | 435 (44) | 160 (16) | 2,286 | | 573 (25) | 1,340 (59) | 337 (15) |
| 2008 | 995 | 376 (38) | 432 (43) | 128 (13) | 2,326 | | 610 (26) | 1,415 (61) | 333 (14) |
| 2009 | 928 | 373 (40) | 375 (40) | 131 (14) | 2,373 | | 609 (26) | 1,437 (61) | 359 (15) |
| 2010 | 1013 | 364 (36) | 387 (38) | 176 (17) | 2,438 | | 637 (26) | 1,457 (60) | 356 (15) |
| 2011 | 1,016 | 407 (40) | 426 (42) | 172 (17) | 2,510 | | 637 (25) | 1,575 (63) | 433 (17) |
| 2012 | 1,035 | 392 (38) | 461 (45) | 194 (19) | 2,414 | | 611 (25) | 1,435 (59) | 411 (17) |
| 2013 | 1,171 | 442 (38) | 494 (42) | 201 (17) | 2,774 | | 668 (24) | 1,741 (63) | 473 (17) |
| 2014 | 1,205 | 441 (37) | 503 (42) | 207 (17) | 2,648 | | 664 (25) | 1,683 (64) | 462 (17) |
| 2015 | 1,271 | 456 (36) | 513 (40) | 203 (16) | 2,812 | | 673 (24) | 1,811 (64) | 508 (18) |
| 2016 | 1,268 | 445 (35) | 547 (43) | 211 (17) | 2,852 | | 769 (27) | 1,844 (65) | 527 (18) |
| 2017 | 1,273 | 440 (35) | 564 (44) | 275 (22) | 2,903 | | 733 (25) | 1,887 (65) | 539 (19) |
| 2018 | 1,309 | 453 (35) | 575 (44) | 235 (18) | 3,100 | | 804 (26) | 2,112 (68) | 599 (19) |
| 2019 | 1,144 | 377 (33) | 496 (43) | 229 (20) | 2,963 | | 744 (25) | 2,013 (68) | 585 (20) |
| **TOTAL** | 15,581 | 5758 (37) | **6,603 (42)** | **2,665 (17)** | 36,620 | | 9265 (25) | **23,100 (63)** | **6,191 (17)** |

Table S2 Baseline characteristics. Men only.

| **Characteristic** | **Renal cell carcinoma** | **Bladder cancer** |
| --- | --- | --- |
| Number of individuals | 9,823 | 27,355 |
| Total number of person-years after cancer index date | 121,067 | 332,536 |
| Follow up years, min (max) | 1 (15) | 1 (15) |
| Follow up years, mean (SD) | 5.5 (3.8) | 5.5 (3.7) |
| Follow up years, median (Q25, Q75) | 5 (2, 8) | 5 (2, 8) |
| Age at cancer diagnosis |  |  |
| Mean (SD) | 66.3 (11.7) | 73.2 (10.6) |
| Median (25th percentile, 75th percentile) | 67.6 (59.4, 74.5) | 73.9 (66.9, 80.7) |
| Primary tumor at diagnosis (T), (%) |  |  |
| T1 | 5,262 (54) | 9 (<1) |
| T2+T3+T4 | 3,595 (37) | 5,164 (19) |
| Ta+Tis | 3 (<1) | 13,406 (49) |
| Tx | 232 (2) | 403 (1) |
| Missing | 731 (7) | 8,373 (31) |
| Regional lymph nodes at diagnosis (N), n (%) |  |  |
| N0 | 6,990 (71) | 13,312 (49) |
| N1+N2+N3 | 891 (9) | 802 (3) |
| Nx | 0 (0) | 0 (0) |
| Missing | 1,942 (20) | 13,241 (48) |
| Distant metastasis at diagnosis (M), n (%) |  |  |
| M0 | 6,898 (70) | 14,368 (53) |
| M1 | 1,616 (16) | 793 (3) |
| Mx | 756 (8) | 10,768 (39) |
| Missing | 553 (6) | 1,426 (5) |
| Diabetes before cancer diagnosis, n (%) | 1,768 (18) | 5,035 (18) |
| Number of individuals with diabetes at any time (%) | 2,484 (25) | 6,682 (24) |
| Steroid treatment one year before cancer, n (%)**^a^** | 1,002 (10) | 2,417 (9) |
| Steroid treatment at any time 1st July 2005-2020 | 5,031 (51) | 12,042 (44) |
| Registered date of death before study end, n (%) | 4,182 (43) | 13,020 (48) |
| Cause of death cancer, n (%) | 2,911 (30) | 7,647 (28) |
| Cause of death other, n (%) | 1,271 (13) | 5,373 (20) |

1. Since we lack drug data before 1 July 2005, this figure is based on persons diagnosed with cancer on or after 1 July 2006. It is not assumed that this restriction will affect he results.

Table S3 Baseline characteristics. Women only.

| **Characteristic** | **Renal cell carcinoma** | **Bladder cancer** |
| --- | --- | --- |
| Number of individuals | 5,758 | 9,265 |
| Total number of person-years after cancer index date | 71,119 | 113,689 |
| Follow up years, min (max) | 1 (15) | 1 (15) |
| Follow up years, mean (SD) | 5.8 (3.9) | 5.4 (3.9) |
| Follow up years, median (Q25, Q75) | 5 (2, 9) | 4 (2, 8) |
| Age at cancer diagnosis |  |  |
| Mean (SD) | 68.3 (11.9) | 73.5 (11.5) |
| Median (25th percentile, 75th percentile) | 69.8 (61.4, 76.8) | 74.5 (66.3, 81.9) |
| Primary tumor at diagnosis (T), (%) |  |  |
| T1 | 3,143 (55) | 6 (<1) |
| T2+T3+T4 | 2,046 (36) | 2,088 (23) |
| Ta+Tis | 0 (0) | 4,447 (48) |
| Tx | 125 (2) | 156 (2) |
| Missing | 444 (8) | 2,568 (28) |
| Regional lymph nodes at diagnosis (N), n (%) |  |  |
| N0 | 4,172 (72) | 4,551 (49) |
| N1+N2+N3 | 438 (8) | 338 (4) |
| Nx | 0 (0) | 1 (<1) |
| Missing | 1,148 (20) | 4,375 (47) |
| Distant metastasis at diagnosis (M), n (%) |  |  |
| M0 | 4,020 (70) | 4,834 (52) |
| M1 | 851 (15) | 399 (4) |
| Mx | 523 (9) | 3,554 (38) |
| Missing | 364 (6) | 478 (5) |
| Diabetes before cancer diagnosis, n (%) | 897 (16) | 1,156 (12) |
| Number of individuals with diabetes at any time (%) | 1,268 (22) | 1,597 (17) |
| Steroid treatment one year before cancer, n (%)**^a^** | 747 (13) | 1,021 (11) |
| Steroid treatment at any time 1st July 2005-2020 | 3,261 (57) | 4,463 (48) |
| Registered date of death before study end, n (%) | 2,467 (43) | 4,363 (47) |
| Cause of death cancer, n (%) | 1,715 (30) | 2,846 (31) |
| Cause of death other, n (%) | 752 (13) | 1,517 (16) |

1. Since we lack drug data before 1 July 2005, this figure is based on persons diagnosed with cancer on or after 1 July 2006. It is not assumed that this restriction will affect he results.

Table S4 Individuals categorised with type 2 diabetes and with diabetes diagnosis after cancer diagnosis.

|  | **Renal cell carcinoma** | **Bladder cancer** |
| --- | --- | --- |
| Number of individuals | 730 | 1,689 |
| Women, n (%) | 251 (34) | 366 (22) |
| Men, n (%) | 479 (66) | 1,323 (78) |
| Days from cancer diagnosis to diabetes diagnosis |  |  |
| Mean (SD) | 1,412 (1,196) | 1,293 (1,147) |
| Median (25th percentile; 75th percentile) | 1,128 (398; 2,149) | 1,003 (349; 1,966) |
| Individuals with insulin treatment, n (%) | 106 (15) | 145 (8) |
| Days from diabetes diagnosis to insulin treatment**^a^** |  |  |
| Mean (SD) | 1,320 (701) | 1,507 (990) |
| Median (25th percentile; 75th percentile) | 1,216 (708; 1,860) | 1,217 (733; 2,069) |

1. 7 individuals with RCC and 6 individuals with BC have registered insulin treatment before defined diabetes diagnosis and not included here.

Table S5 Number of glycaemic events for people with RCC and diabetes, controls with diabetes but no RCC, and for RCC without diabetes. Results by 1,000 individuals and by 10,000 person years. All events and period after cancer index date and all events after diabetes index date.

| **Statistic** | **Unit** | **RCC with diabetes^a^** | **Control with diabetes^b^** | **p-value** | **RCC without diabetes^a^** |
| --- | --- | --- | --- | --- | --- |
| **All events 2006-2020** | | | | | |
| RCC study group | n (person-years) | 2,660 (31,187) | 12,742 (161,899) |  | 11,829 (146,520) |
| Total number of visits or hospitalisations (main diagnosis only) | # per 1,000 individuals (#) | 101.9 (271) | 86.9 (1,115) | <0.001 | 0.9 (11) |
|  | # per 10,000 person years (#) | 86.9 (271) | 68.9 (1,115) | <0.001 | 0.8 (11) |
| Total number of visits or hospitalisations (main or secondary diagnosis) | # per 1,000 individuals (#) | 156.4 (416) | 122.3 (1,558) | <0.001 | 3.8 (45) |
|  | # per 10,000 person years (#) | 133.4 (416) | 96.2 (1,558) | <0.001 | 3.1 (45) |
| ≥1 visit or admission (main or secondary diagnosis) | n per 1,000 individuals (n) | 91.0 (242) | 75.3 (960) | 0.007 | 3.2 (38) |
| ≥1 filled prescription of steroids after cancer diagnosis and ≥1 visit or admission for malglycemia (main or secondary diagnosis) | n per 1,000 individuals (n) | 41.7 (111) | 21.8 (278) | <0.001 | 2.5 (29) |
| **Events 2006-2020 after diabetes index date** | | | | | |
| Total number of visits or hospitalisations (main diagnosis only) | # per 1,000 individuals (#) | 100 (266) | 85.5 (1,089) | <0.001 |  |
|  | # per 10,000 person years (#) | 103.6 (266) | 82.0 (1,089) | <0.001 |  |
| Total number of visits or hospitalisations (main or secondary diagnosis) | # per 1,000 individuals (#) | 152.6 (406) | 117.3 (1,494) | <0.001 |  |
|  | # per 10,000 person years (#) | 158.1 (406) | 112.5 (1,494) | <0.001 |  |
| ≥1 visit or admission (main or secondary diagnosis) | n per 1,000 individuals (n) | 88.0 (234) | 71.5 (911) | 0.004 |  |
| ≥1 filled prescription of steroids after cancer diagnosis and ≥1 visit or admission for malglycemia (main or secondary diagnosis) | n per 1,000 individuals (n) | 41.0 (109) | 21.0 (267) | <0.001 |  |
| **Events 2006-2020 after cancer index date** | | | | | |
| Total number of visits or hospitalisations (main diagnosis only) | # per 1,000 individuals (#) | 66.6 (177) | 57.9 (649) | <0.001 | 0.9 (11) |
|  | # per 10,000 person years (#) | 132.0 (177) | 87.9 (649) | <0.001 | 1.7 (11) |

1. Only include people with RCC and BC with registered diabetes diagnosis before cancer index date. Note that the number of persons differ from Table 2. This is due to that there are persons that migrate before they receive their cancer diagnosis and thus are excluded in the analysis for the study population after cancer diagnosis.
2. Note that the number of persons differ from Table 2. This is due to that there are persons that migrate and die before their cases receive their cancer diagnosis and thus are excluded in the analysis for the study population after cancer diagnosis.

Table S6 Number of glycemic events for people with BC and diabetes, controls with diabetes but no BC, and for BC without diabetes. Results by 1,000 individuals and by 10,000 person years. All events and period after cancer index date and all events after diabetes index date.

| **Statistic** | **Unit** | **BC with diabetes^a^** | **Control with diabetes^b^** | **p-value** | **BC without diabetes^a^** |
| --- | --- | --- | --- | --- | --- |
| **All events 2006-2020** | | | | | |
| BC study group | n (person-years) | 6,178 (70,786) | 29,054 (341,723) |  | 28,341 (347,621) |
| Total number of visits or hospitalisations (main diagnosis only) | # per 1,000 individuals (#) | 97.0 (599) | 72.0 (2,091) | <0.001 | 1.1 (32) |
|  | # per 10,000 person years (#) | 84.6 (599) | 61.2 (2,091) | <0.001 | 0.9 (32) |
| Total number of visits or hospitalisations (main or secondary diagnosis) | # per 1,000 individuals (#) | 141.6 (875) | 105.5 (3,066) | <0.001 | 3.6 (102) |
|  | # per 10,000 person years (#) | 123.6 (875) | 89.7 (3,066) | <0.001 | 2.9 (102) |
| ≥1 visit or admission (main or secondary diagnosis) | n per 1,000 individuals (n) | 86.4 (599) | 68.3 (1985) | <0.001 | 2.9 (82) |
| ≥1 filled prescription of steroids after cancer diagnosis and ≥1 visit or admission for malglycemia (main or secondary diagnosis) | n per 1,000 individuals (n) | 31.9 (197) | 17.6 (510) | <0.001 | 1.1 (30) |
| **Events 2006-2020 after diabetes index date** | | | | | |
| Total number of visits or hospitalisations (main diagnosis only) | # per 1,000 individuals (#) | 96.0 (593) | 70.5 (2,047) | <0.001 |  |
|  | # per 10,000 person years (#) | 100.8 (593) | 74.2 (2,047) | <0.001 |  |
| Total number of visits or hospitalisations (main or secondary diagnosis) | # per 1,000 individuals (#) | 137.7 (851) | 100.4 (2,912) | <0.001 |  |
|  | # per 10,000 person years (#) | 144.7 (851) | 105.5 (2,912) | <0.001 |  |
| ≥1 visit or admission (main or secondary diagnosis) | n per 1,000 individuals (n) | 84.2 (520) | 64.2 (1,864) | <0.001 |  |
| ≥1 filled prescription of steroids after cancer diagnosis and ≥1 visit or admission for malglycemia (main or secondary diagnosis) | n per 1,000 individuals (n) | 31.2 (193) | 16.8 (487) | <0.001 |  |
| **Events 2006-2020 after cancer index date** | | | | | |
| Total number of visits or hospitalisations (main diagnosis only) | # per 1,000 individuals (#) | 65.7 (406) | 50.4 (1,118) | <0.001 | 0.8 (23) |
|  | # per 10,000 person years (#) | 135.2 (406) | 84.0 (1,118) | <0.001 | 1.5 (23) |

1. Only include people with RCC and BC with registered diabetes diagnosis before cancer index date. Note that the number of persons differ from Table 2. This is due to that there are persons that migrate before they receive their cancer diagnosis and thus are excluded in the analysis for the study population after cancer diagnosis.
2. Note that the number of persons differ from Table 2. This is due to that there are persons that migrate and die before their cases receive their cancer diagnosis and thus are excluded in the analysis for the study population after cancer diagnosis.

# Supplementary D Result figures

## Glycemic events


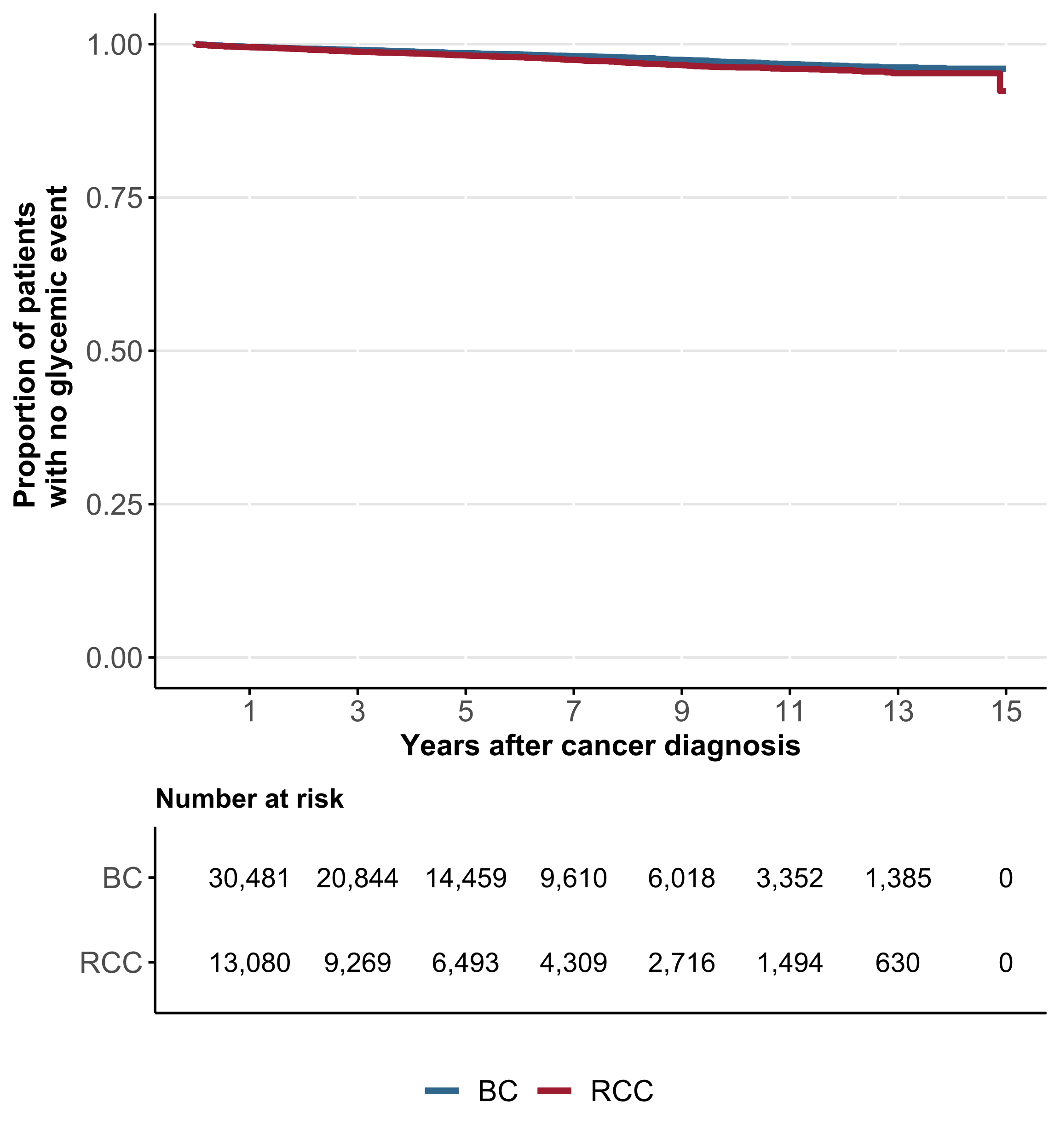


Figure S5 Kaplan Meier estimation of time to first glycemic event leading to a hospital visit or admission after diagnosis of BC and RCC with and without diabetes.

Log rank test: p = 0.001.


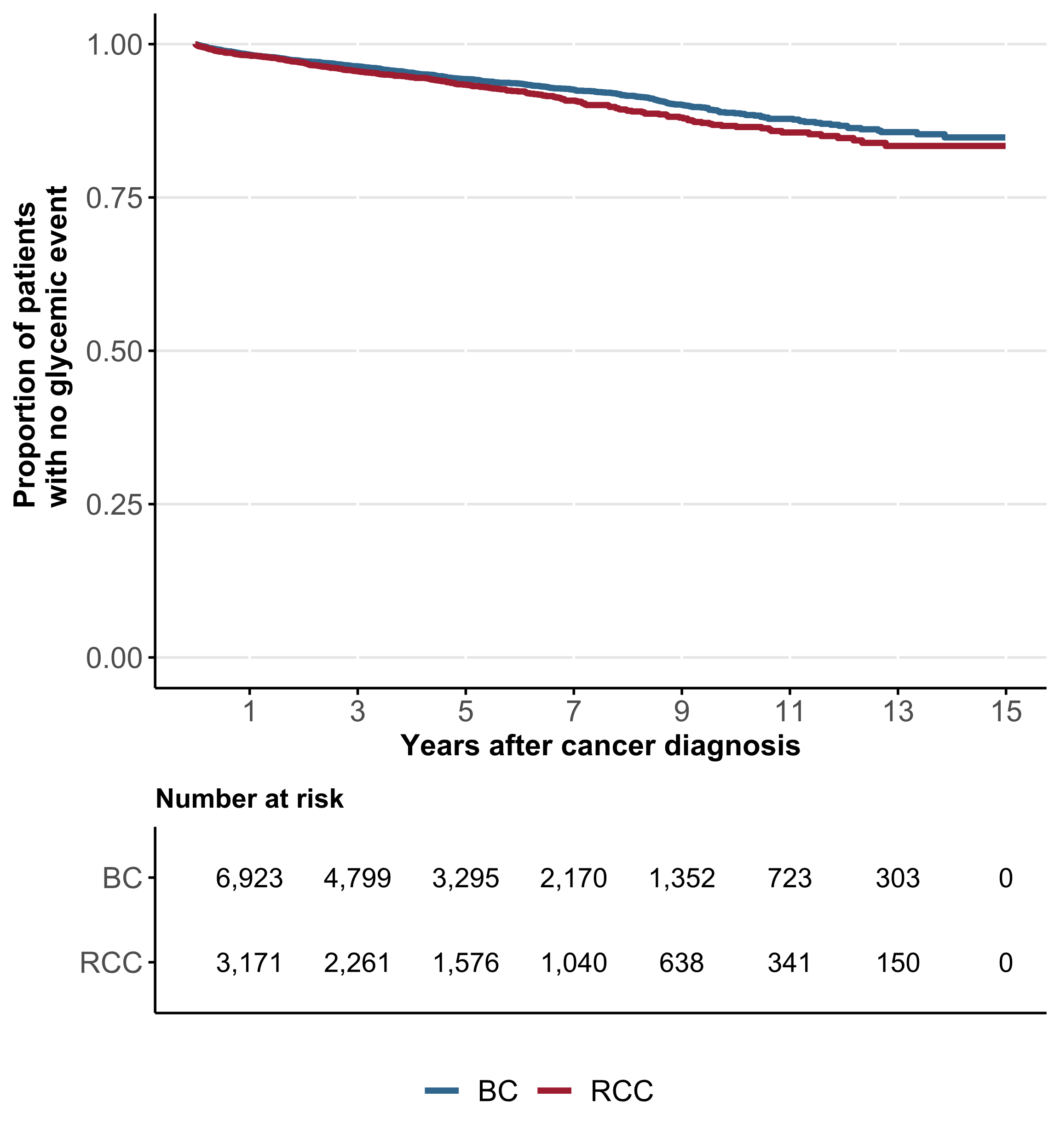


Figure S6 Kaplan Meier estimation of time to first glycemic event leading to a hospital visit or admission from diagnosis of BC and RCC with diabetes


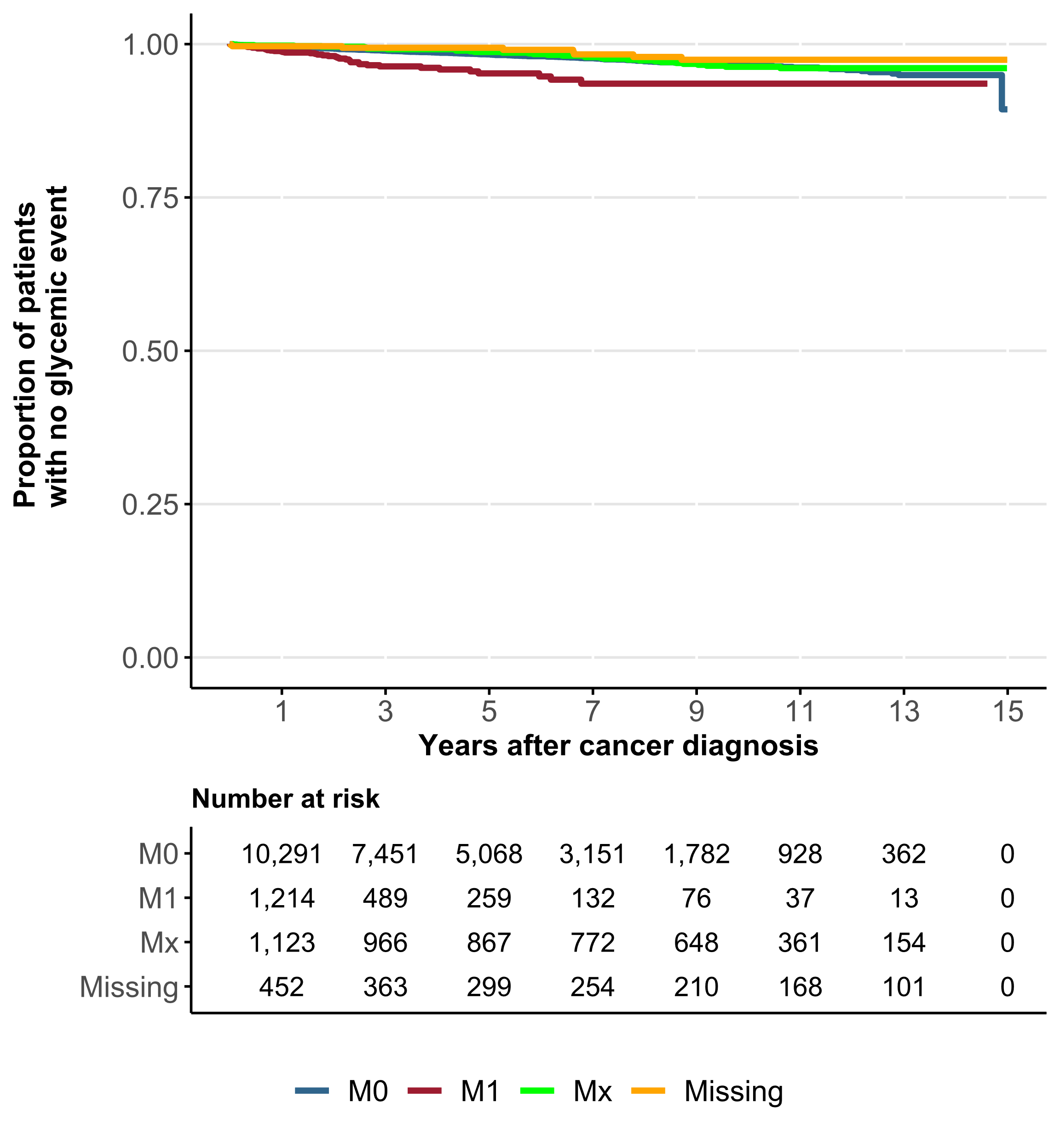


Figure S7 Kaplan Meier estimation of time to first glycaemic event leading to a hospital visit or admission after diagnosis of RCC stratified by status for metastasis at diagnosis.

Log rank test: p = <0.001.


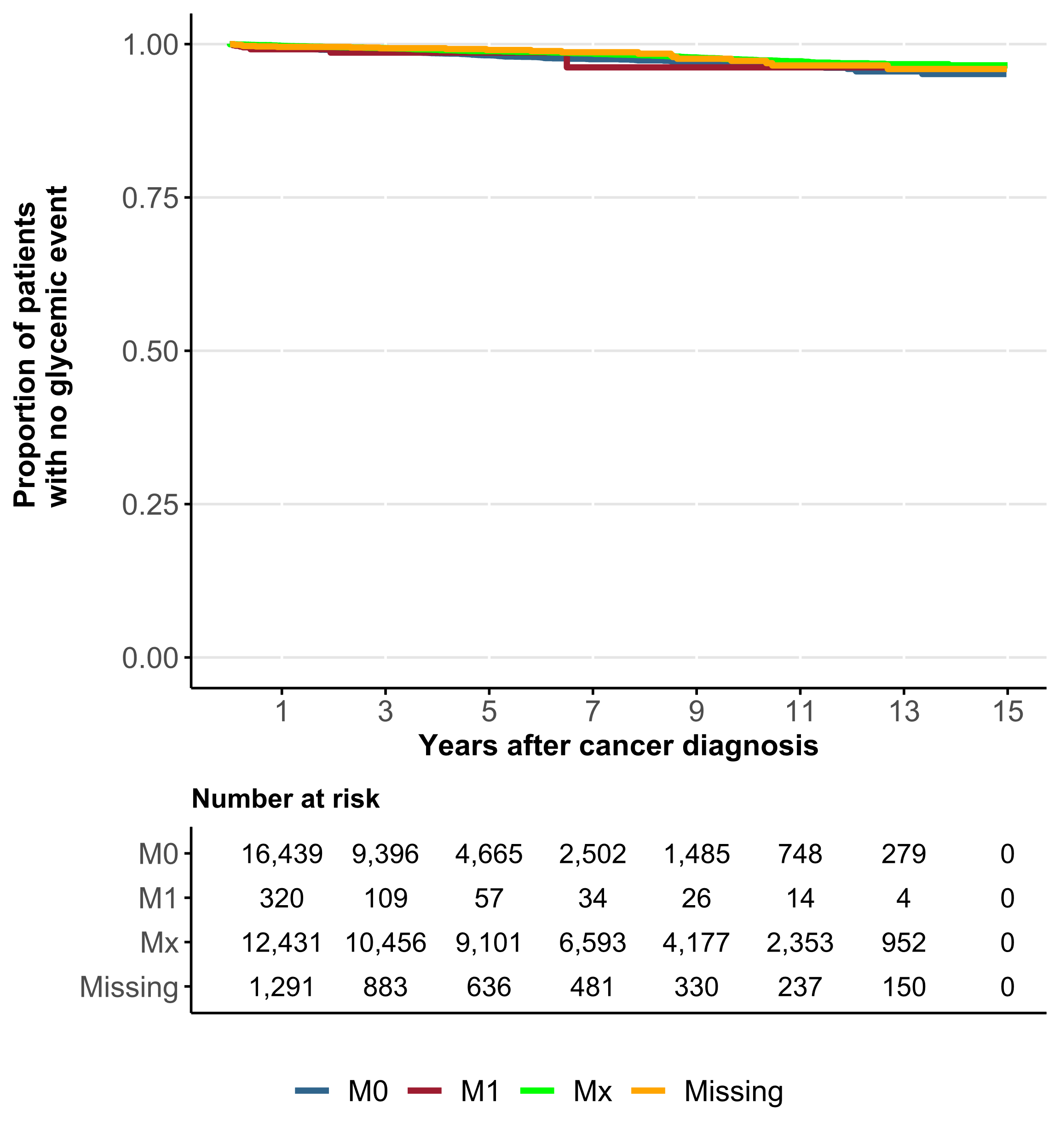


Figure S8 Kaplan Meier estimation of time to first glycaemic event leading to a hospital visit or admission after diagnosis of BC stratified by status for metastasis at diagnosis.

Log rank test: p = <0.001.


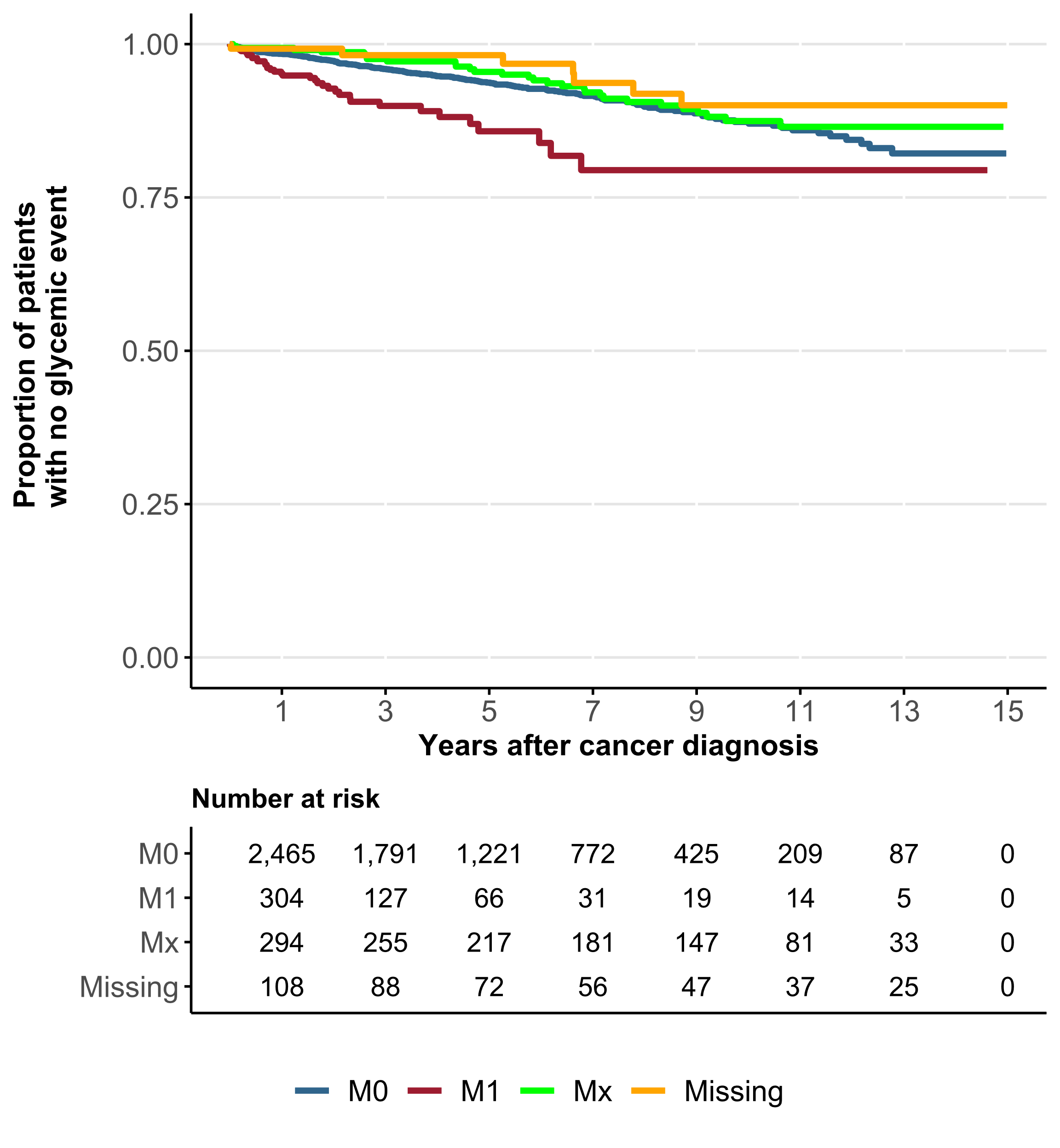


Figure S9 Kaplan Meier estimation of time to first glycaemic event leading to a hospital visit or admission from diagnosis of RCC with diabetes stratified by status for metastasis at diagnosis.

Log rank test: p = <0.001.


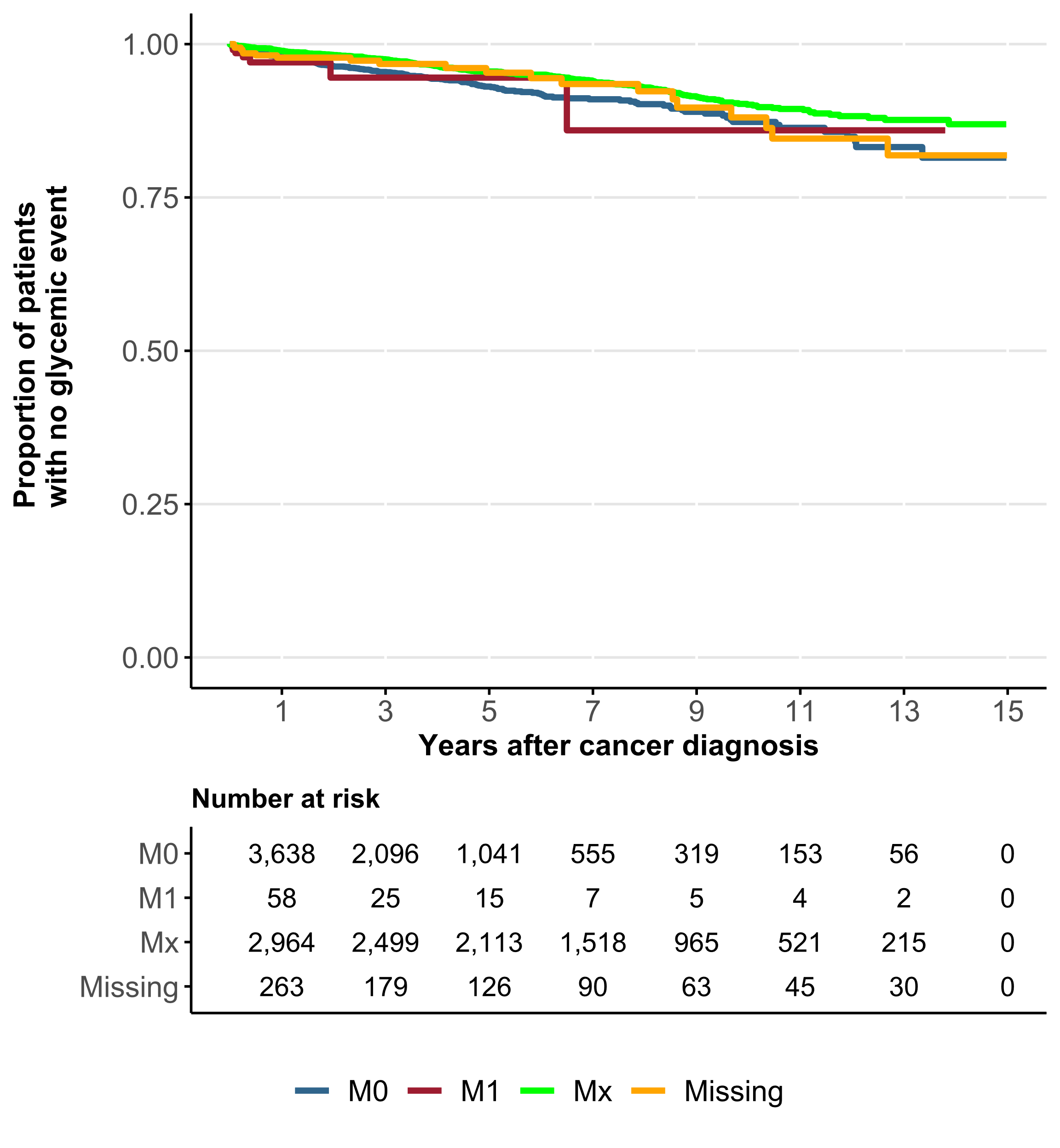


Figure S10 Kaplan Meier estimation of time to first glycaemic event from diagnosis of BC with diabetes stratified by status for metastasis at diagnosis.

Log rank test: p = <0.001.

## Filled prescriptions of steroids


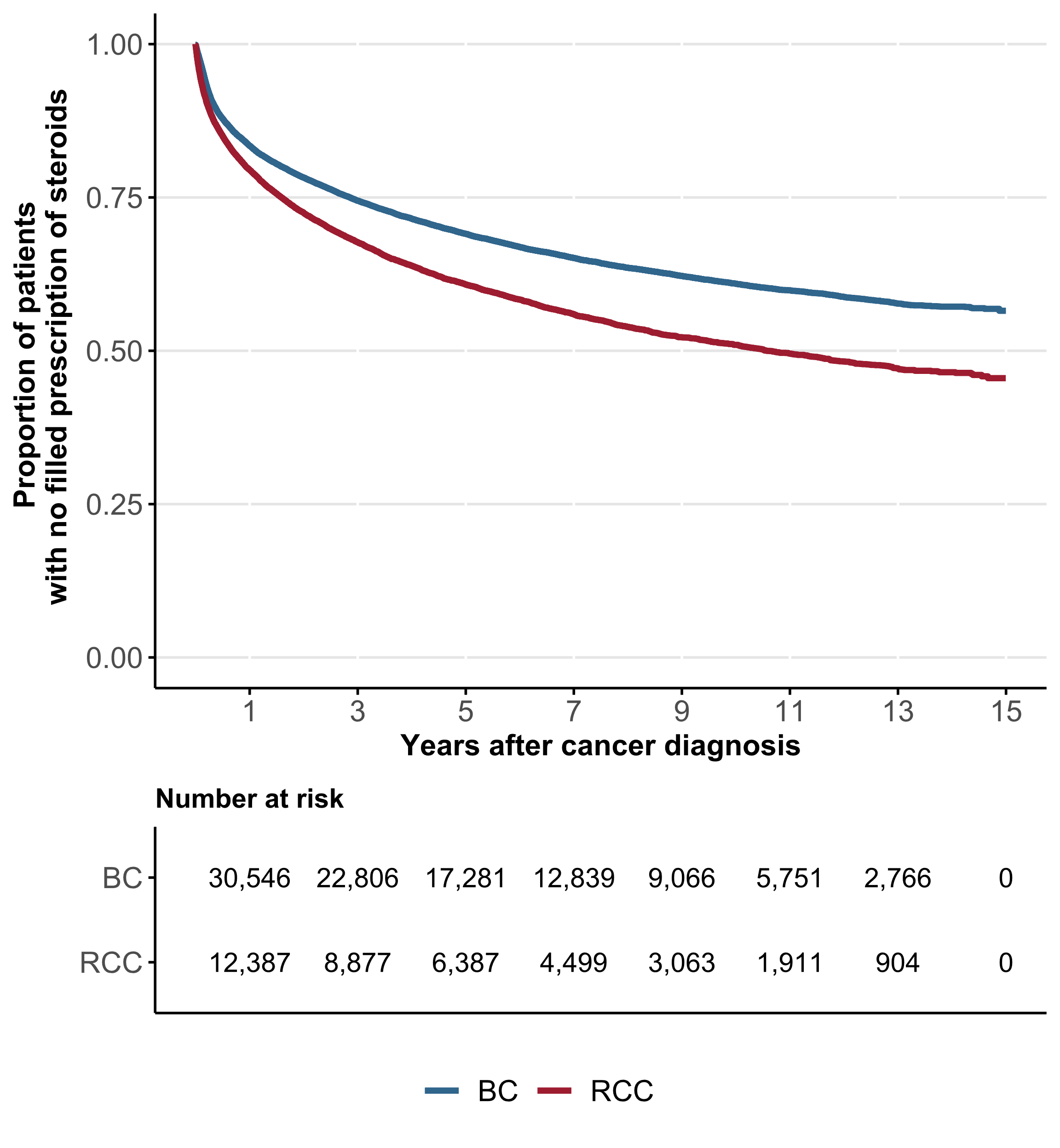


Figure S11 Time to first filled prescription of steroids after diagnosis of BC and RCC.

Log rank test: p = <0.001.


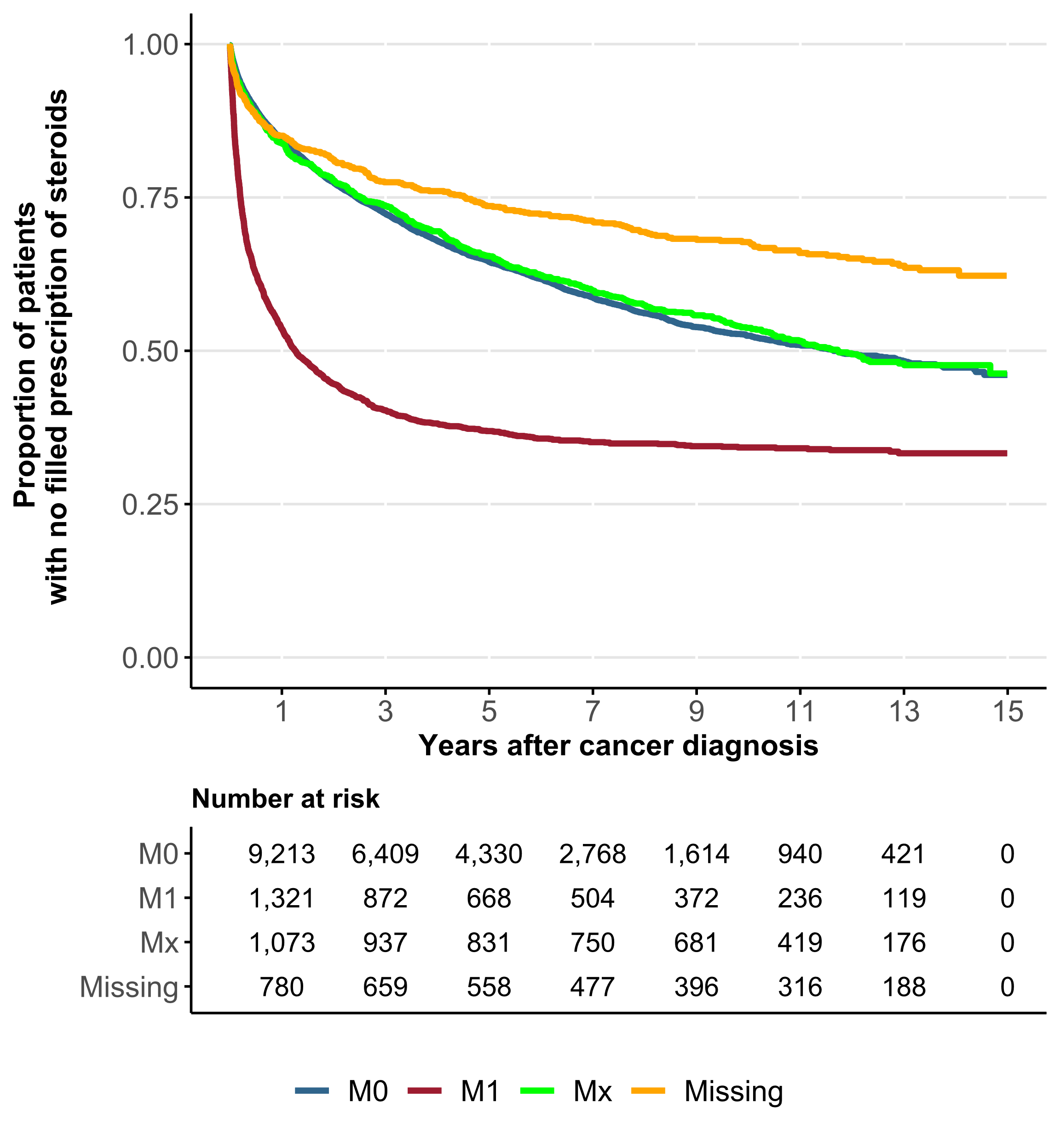


Figure S12 Kaplan Meier estimation of time to first filled prescription of steroids from diagnosis of RCC stratified by registered M-status according to TNM staging system on cancer index date.

Log rank test: p = <0.001.


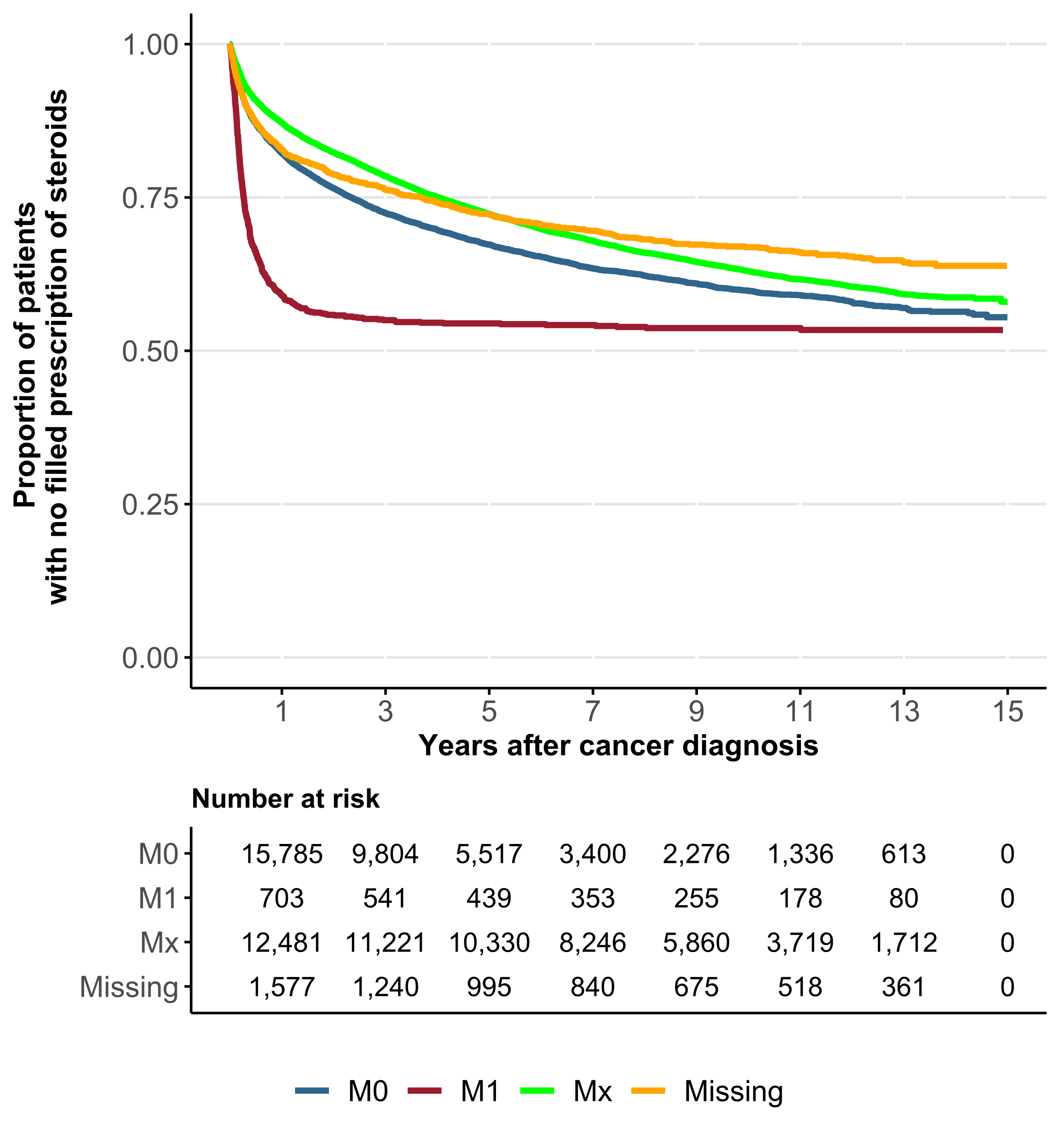


Figure S13 Kaplan Meier estimation of time to first filled prescription of steroids from diagnosis of BC stratified by registered status of radiologically shown metastasis on cancer index date.

## Insulin treatment

Figures S14-S17 presents survival time analyses for RCC and BC of time to start of insulin treatment for people with cancer and type 2 compared to people with type 2 diabetes controls but no cancer. While Figure 3 and 4 in the main text applies the cancer index date as start of observation, Figures S14-S17 anchor the analyses in start of diabetes with two alternative definitions of first observed diabetes indication 1) 1 January 1997 or later (Figures S14 and S15); and 2) 1 July 2005 or later (Figures S16 and S17). The data from the National Patient Register are available from 1997 and are expected to capture people with type 2 diabetes and need for hospital-based care, for instance because of complications. The data from the National Prescribed Drug Register are available from July 2005 and implies a broader coverage of people with type 2 diabetes as soon as they start using glucose lowering medications. The second definition implies a truncation of the diabetes history ignoring diabetes onset history before July 2005. Controls with type 2 diabetes but no cancer are matched on age, sex and the same year of diabetes diagnosis.

Figure S14 Kaplan-Meier survival curves for start of insulin treatment for people with type 2 diabetes and later RCC diagnosis compared to matched controls with type 2 diabetes only. Analysis time from first observed diabetes after 1 January 1997. RCC diagnosis from Jan 1, 2006 to Dec

Figure S15 Kaplan-Meier survival curves for start of insulin treatment for people with type 2 diabetes and later BC diagnosis compared to matched controls with type 2 diabetes only. Analysis time from first observed diabetes after 1 January 1997. RCC diagnosis from Jan 1, 2006 to Dec

Figure S16 Kaplan-Meier survival curves for start of insulin treatment for people with diagnosis of RCC and type 2 diabetes before cancer diagnosis. Controls have same year of diabetes diagnosis as cancer cases. Observation time starts Jul 1, 2005.

Log-rank test p<0.001.

Figure S17 Kaplan-Meier survival curves for start of insulin treatment for people with diagnosis of BC and type 2 diabetes before cancer diagnosis. Controls have same year of diabetes diagnosis as cancer cases. Observation time starts Jul 1, 2005.

Log-rank test p<0.001.
